# An integrative *in-silico approach* for therapeutic target identification in the human pathogen *Corynebacterium diphtheriae*

**DOI:** 10.1371/journal.pone.0186401

**Published:** 2017-10-19

**Authors:** Syed Babar Jamal, Syed Shah Hassan, Sandeep Tiwari, Marcus V. Viana, Leandro de Jesus Benevides, Asad Ullah, Adrián G. Turjanski, Debmalya Barh, Preetam Ghosh, Daniela Arruda Costa, Artur Silva, Richard Röttger, Jan Baumbach, Vasco A. C. Azevedo

**Affiliations:** 1 PG program in Bioinformatics (LGCM), Institute of Biological Sciences, Federal University of Minas Gerais, Belo Horizonte, MG, Brazil; 2 Department of Chemistry, Islamia College University Peshawar, KPK, Pakistan; 3 Departamento de Química Biológica, Facultad de Ciencias Exactas y Naturales, Universidad de Buenos Aires, Pabellón II, Buenos Aires, Argentina; 4 Centre for Genomics and Applied Gene Technology, Institute of Integrative Omics and Applied Biotechnology, Nonakuri, Purba Medinipur, West Bengal, India; 5 Department of Computer Science, Virginia Commonwealth University, Richmond, VA, United States of America; 6 Institute of Biologic Sciences, Federal University of Para, Belém, PA, Brazil; 7 Department of Mathematics and Computer Science, University of Southern Denmark, Odense, Denmark; 8 Department of General Biology (LGCM), Institute of Biologic Sciences, Federal University of Minas Gerais, Belo Horizonte, MG, Brazil; UMR-S1134, INSERM, Université Paris Diderot, INTS, FRANCE

## Abstract

*Corynebacterium diphtheriae* (Cd) is a Gram-positive human pathogen responsible for diphtheria infection and once regarded for high mortalities worldwide. The fatality gradually decreased with improved living standards and further alleviated when many immunization programs were introduced. However, numerous drug-resistant strains emerged recently that consequently decreased the efficacy of current therapeutics and vaccines, thereby obliging the scientific community to start investigating new therapeutic targets in pathogenic microorganisms. In this study, our contributions include the prediction of modelome of 13 *C*. *diphtheriae* strains, using the MHOLline workflow. A set of 463 conserved proteins were identified by combining the results of pangenomics based core-genome and core-modelome analyses. Further, using subtractive proteomics and modelomics approaches for target identification, a set of 23 proteins was selected as essential for the bacteria. Considering human as a host, eight of these proteins (glpX, nusB, rpsH, hisE, smpB, bioB, DIP1084, and DIP0983) were considered as essential and non-host homologs, and have been subjected to virtual screening using four different compound libraries (extracted from the ZINC database, plant-derived natural compounds and Di-terpenoid Iso-steviol derivatives). The proposed ligand molecules showed favorable interactions, lowered energy values and high complementarity with the predicted targets. Our proposed approach expedites the selection of *C*. *diphtheriae* putative proteins for broad-spectrum development of novel drugs and vaccines, owing to the fact that some of these targets have already been identified and validated in other organisms.

## Introduction

*Corynebacterium diphtheriae* is responsible for causing diphtheria which remains a major global cause of death (http://www.who.int/immunization_monitoring/diseases/diphteria/), and has conventionally been divided into four subgroups of biovars *i*.*e*., gravis, intermedius, mitis and belfanti based on biochemical characteristics according to Funke *et al*., 1997 [1] and Whitman *et al*., 2012 [2]. It was once a major cause of infant mortality, which spread as an epidemic and resulted in thousands of deaths [3]. The death rates dropped over time specifically in countries where living standards have improved, and the death rates rapidly declined after the introduction of immunization programs [3]. Despite these measures, it remains a significant pathogen around the globe, even today. A variety of mechanisms were responsible for causing such death rates; for example the ‘strangling angel’ effect on children that ascended from the wing shaped pseudo-membranes formed in the oropharynx. Disarticulation and impaction of these pseudo-membranes triggers acute airway obstruction and can result in sudden death [3, 4]. Since there has been a plethora of reported cases on both non-lethal and lethal diphtheria across various countries in the past few years, and that significant population displacements in the form of immigration are happening, more such cases are bound to follow. A passable handling requires quick inroads in discovering diphtheria antitoxin and antibiotic treatment [5].

Computational methods and other approaches, like reverse vaccinology, have been established for the rapid identification of novel targets in the post-genomic era [6, 7]. Approaches like subtractive and comparative microbial genomics as well as differential genome analysis [8] are being used for the identification of targets in a number of human pathogens like *M. tuberculosis [9]*, *Burkholderia pseudomalleii* [10], *Helicobacter pylori* [11] *Pseudomonas aeruginosa* [12], *Neisseria gonorrhea* [13] and *Salmonella typhi* [14].

The main principle is to find targeted genes/proteins that are essential for the pathogen and possess no homology counterpart in the host [15], such that drugs targeting these “pathogen-essential non-host homologs” can be applied with little (or no) off targets in the host. Some pathogen-essential proteins, though, may possess a certain degree of homology to host proteins. However, they might still be selected as potential molecular targets for structure-based selective inhibitor development. Significant differences in the active sites or in other druggable pockets might exist, such that the pathogenic protein could still be targeted [16, 17].

Here, we exploit an integrative *in silico* approach for the predictive proteome of *C*. *diphtheriae* species to associate the genomic information with the identification of putative therapeutic targets based on their three dimensional structure. It can be utilized for the identification of potent inhibitors, which might possibly lead to the discovery of compounds that inhibit pathogenic growth. The predicted proteomes from the 13 genomes of C. *diphtheriae* were modeled (pan-modelome) using the MHOLline workflow as proposed by Hassan *et al*., 2014 [18]. Furthermore, intra-species conserved proteins with adequate 3D models (core-modelome) were filtered on the basis of predicted essentiality for the bacteria, which leads to the identification of eight essential bacterial proteins. They were found non homologous to all host proteins and have been subjected to virtual screening using multiple compound libraries.

We provided a list of putative targets in *C*. *diphteriae*, and possible mechanisms to design peptide vaccines, and suggest novel lead, natural and drug-like compounds that could bind to the proposed target proteins.

## Materials and methods

### Genomes selection

**Fig 1:**
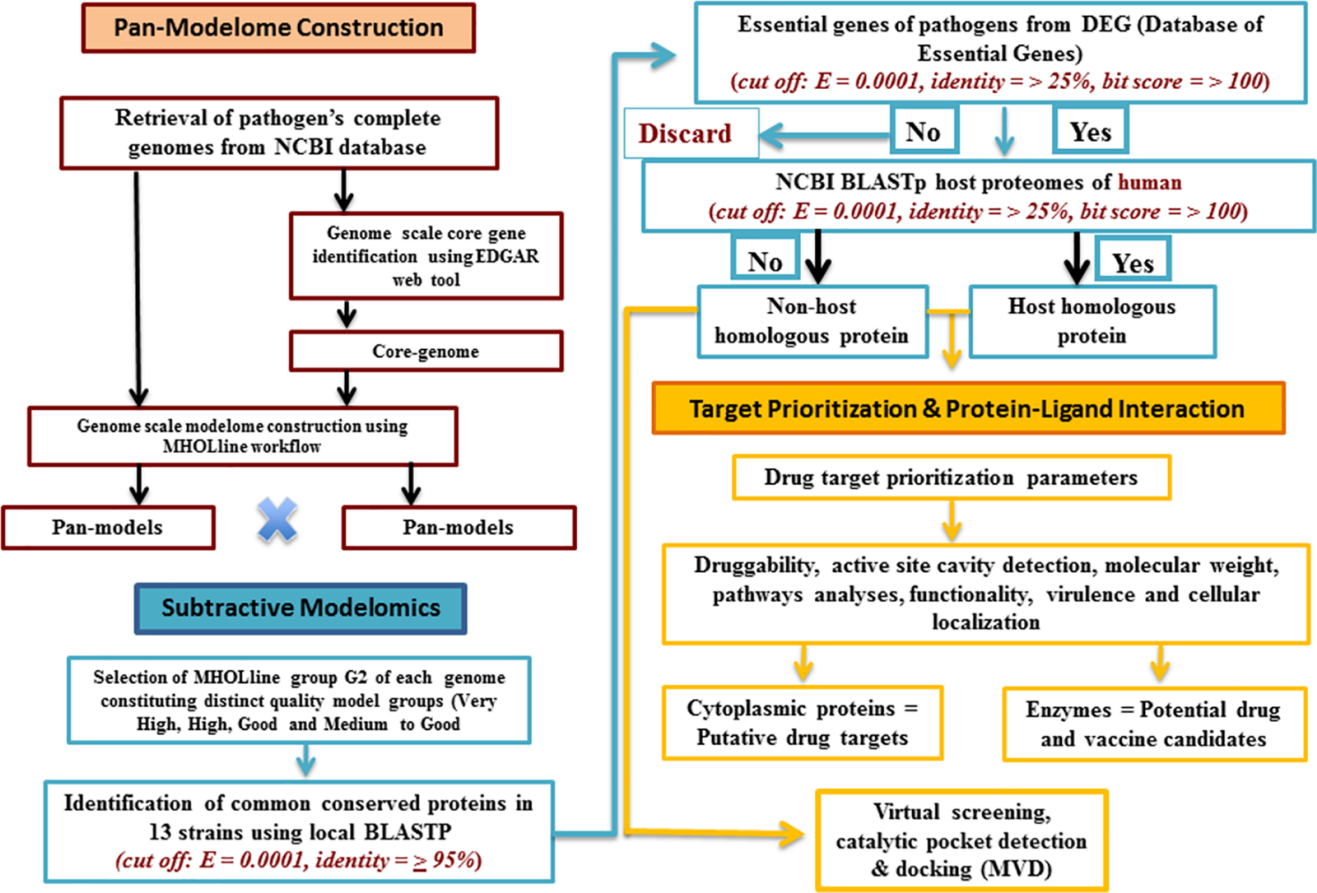
Overview of different computational steps employed for the identification of putative essential targets (non-host homologous and host homologous) from the core-proteome of 13 *C*. *diphtheriae* strains.

**Fig 2:**
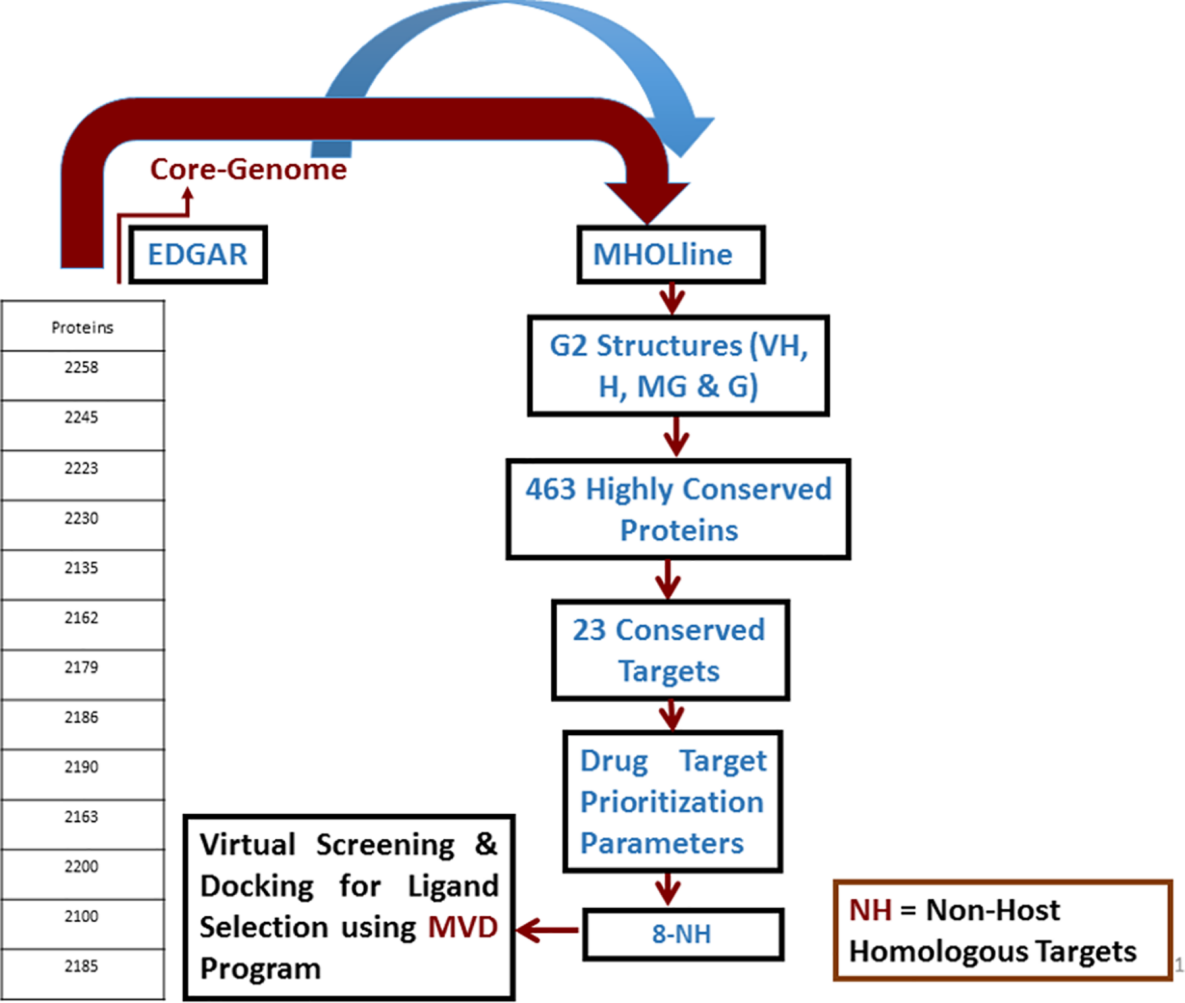
Intra-species subtractive modelomics workflow for conserved target identification in *C*. *diphtheriae* species. The table represents the total number of protein sequences as an input data fed to the MHOLline workflow (upper red arrow). The blue arrow represents the core genes of thirteen Cd strains. The rectangular boxes show how this workflow processes and filters a large quantity of genomic data for putative drug and vaccine target identification of a pathogen.

The thirteen *C*. *diphtheriae* strains, including three of the four biovars: gravis, mitis and belfanti **(Table 1)** were included in this study. The gene and protein sequences of these thirteen *C*. *diphtheriae* strains were retrieved from NCBI (ftp://ftp.ncbi.nih.gov/genomes/Bacteria). The different steps involved in this computational approach for genome-scale modelome prediction and for the prioritization of putative drug and vaccine targets are given in (**Figs 1 & 2**).

**Table 1:** Strains of *C*. *diphtheriae* employed in the pan-modelome study with information on genomes statistics, disease prevalence and location of isolation.

Strain	GPID	NCBI Accession	Genome Size (Mb)	Proteins	GC%	Location	Biovar
31A	PRJNA42399	NC_016799.1	2.53535	2258	53.60	Brazil	—
241	PRJNA42407	NC_016782.1	2.42655	2245	53.40	Brazil	—
BH8	PRJNA42423	NC_016800.1	2.48552	2223	53.60	Brazil	—
C7	PRJNA42401	NC_016801.1	2.49919	2230	53.50	USA	—
CDCE8392	PRJNA42405	NC_016785.1	2.43333	2135	53.60	USA	Mitis
HC01	PRJNA42409	NC_016786.1	2.42715	2162	53.40	Brazil	Mitis
HC02	PRJNA42411	NC_016802.1	2.46861	2179	53.70	Brazil	Mitis
HC03	PRJNA42415	NC_016787.1	2.47836	2186	53.50	Brazil	Mitis
HC04	PRJNA42417	NC_016788.1	2.48433	2190	53.50	Brazil	Gravis
INCA402	PRJNA42419	NC_016783.1	2.44907	2163	53.70	Brazil	Belfanti
PW8	PRJNA42403	NC_016789.1	2.53068	2200	53.70	USA	—
VA01	PRJNA42421	NC_016790.1	2.39544	2100	53.40	Brazil	Gravis
NCTC13129	PRJNA87	NC_002935.2	2.48863	2185	53.50	UK	Gravis

### Prediction of core-modelome and identification of core genome

To construct the core-modelome of *C*. *diphtheriae*, we followed a slightly modified protocol described by Hassan *et al*., 2014 [18]. High throughput structural modeling, MHOLline (http://www.mholline.lncc.br), was used to predict the modelome (whole-proteome set of protein 3D models) for each strain. MHOLline uses comparative modeling approach for protein 3D structure prediction through MODELLER [19]. Our workflow also includes BLASTp (Basic Local Alignment Search Tool for Protein) [20], HMMTOP (Prediction of transmembrane helices and topology of proteins), [21] BATS (Blast Automatic Targeting for Structures), FILTERS, ECNGet (Get Enzyme Commission Number), MODELLER, and PROCHECK [22].

MHOLline work on the basis of available template. It is probable that MHOLline cannot detect all the common conserved proteins due to the unavailability of the template. To overcome this probability, we used EDGAR (an **E**fficient **D**atabase framework for comparative **G**enome **A**nalyses using BLAST score **R**atios for pan-genomics analysis) to collect common conserved genome as well of all Cd strains [23]. Later, the results from MHOLine and EDGAR were compared and crosschecked to obtain the final dataset of common conserved proteins.

### Identification of intra-species conserved proteins

Primarily, for the identification of highly conserved proteins with available 3D models in all Cd strains (≥ 95% sequence identity), the standalone release of NCBI BLASTp+ (v2.2.26) was adapted from the NCBI ftp. Site (ftp://ftp.ncbi.nlm.nih.gov/blast/executables/blast+/LATEST/) and installed on a local machine. Furthermore, a search was performed using NCTC13129 as a random reference genome for all strains. Comparative genomics/proteomics approach was next adopted for selecting the highly conserved proteins using an all-against-all BLASTp analysis with a cut-off value of *E = 0*.*0001*, as in many other essentiality studies before [6, 13, 15, 18, 24].

### Essential and non-host homologous (ENH) protein targets

A subtractive genomics approach was next followed for the selection of conserved targets, which were essential to the bacteria [15]. Concisely, the set of proteins derived from the core-modelome of *C*. *diphtheriae* was subjected to the Database of Essential Genes (DEG) for homology analyses. The DEG encompasses experimentally validated data of currently available essential genomic elements like protein-coding genes and non-coding RNAs, from bacteria, archaea and eukaryotes. For a bacterium, essential genes form a minimal genome, i.e., a set of functional modules that has key roles in the emerging field of synthetic biology [25]. The cutoff values used for BLASTp were: *E-value = 0*.*0001*, *bit score ≥100* and *identity ≥ 25%* [15, 18].

The pool of essential genes was then subjected to NCBI-BLASTp (*E-value = 0*.*0001*, *bit score ≥100* and *identity ≥ 25%*) against the human genome for filtering pathogen-essential host-homologs [6]. The remaining set of pathogen-essential non-host homologs were additionally crosschecked with NCBI-BLASTp PDB database using the default values to find any remote structural similarity with the existing host homolog protein structures, keeping the cutoff level to ≤ 15% for query coverage. The biochemical pathways of these proteins have been checked using KEGG (Kyoto Encyclopedia of Genes and Genomes) [26], functionality using UniProt (Universal Protein Resource) [27], virulence using PAIDB (Pathogenicity island database) [28], and cellular localization using CELLO (subCELlular LOcalization predictor) [29]. The final list of targets was based on criteria described by Barh *et al*., 2011 & Hassan *et al*., 2014 [15, 18].

### Essential and host homologous (EH) protein targets

We further extended our analyses to the set of protein targets that were essential to *C*. *diphtheriae* but homologous to host proteins. The essential protein targets deviating from the cutoff values for essential non-host homologous proteins were treated as host homologous proteins. This set of targets was also checked for pathway involvement, functional annotation virulence, and cellular localization as mentioned above.

### Computational identification of druggable pockets

The information obtained from 3D structures and druggability analyses are important features for prioritizing and authenticating putative pathogen targets [30, 31]. As mentioned above, for druggability analyses, the final list of essential non-host and host homologous protein targets were subjected to DoGSiteScorer in PDB format [32]. The DoGSiteScorer is an automated pocket detection and analysis tool for calculating the druggability of protein cavities. For each detected cavity the tool returns the pocket residues and a druggability score ranging from 0 to 1. Values closer to 1 indicate highly druggable protein cavity, i.e. the predicted cavities are likely to bind ligands with high affinity [32]. The DoGSiteScorer also calculates volume, depth, surface area, lipophilic surface, and further parameters for each predicted cavity.

### Ligand libraries preparation, virtual screening and docking analyses

The ligand libraries were prepared from four different sources, compounds from ZINC database (ZINC drug-like molecules, ZINC Natural Product), natural compounds from literature survey [33] and the Di-terpenoid Iso-steviol derivatives (**S1 Table**). ZINC (drug-like molecules) contains 11,193 drug-like molecules, with Tanimoto cutoff level of 60% [34] and ZINC (Natural Product) contain 11,203 molecules. Whereas, the small library of natural compounds contained 28 molecules and the library of Di-terpenoid Iso-steviol derivatives contained 31 molecules respectively. The structures of these molecules were constructed using MOE-Builder tool. The 3D structures were modeled and partial charges were calculated using MOE (Molecular Operating Environment). The energies of the modeled molecules were minimized using the energy minimization algorithm of MOE tool (gradient: 0.05, Force Field: MMFF94X, Chiral Constraint) [35]. The modeled molecules were saved in the.mol2 file format and subjected to docking analysis.

The 3D structures of proteins were examined for structural errors such as missing atoms, wrong bonds and protonation states in the MVD (Molegro Virtual Docker) [36]. The consensus set of protein cavities and those predicted with DogSiteScorer (druggability ≥ 0.80) were compared with the MVD detected cavities, for all Cd targets. The maximum numbers of residues from DoGSiteScorer falling in the cavities detected by MVD were merged and final grid was generated based on the consensus between the highest scoring pocket from DoGSiteScorer and cavities detected by MVD for docking. The most druggable cavity was subjected to virtual screening using MVD. The program comprises of three search algorithms for molecular docking analyses namely MolDock Simplex Evolution (SE), MolDock Optimizer [36] and Iterated Simplex (IS). We employed the MolDock Optimizer search algorithm, which is based on a differential evolutionary algorithm, using the default parameters that are **a)** population size = 50, **b)** scaling factor = 0.5 and **c)** crossover rate = 0.9. The orientations of docked molecules from the library of natural compounds and from the derivatives of Di-terpenoid Iso-steviol were analyzed in Chimera [37]. The 200 top ranked compounds (ZINC drug-like molecules, ZINC Natural Product) for each target protein were evaluated for shape complementarity and hydrogen bond interactions. This led to the selection of a final set of compounds with polypharmacology and polypharmacy characteristics for target proteins in *C*. *diphtheriae*.

## Results and discussion

### Modelome prediction and conserved targets identification in *C*. *diphtheriae*

**Fig 3:**
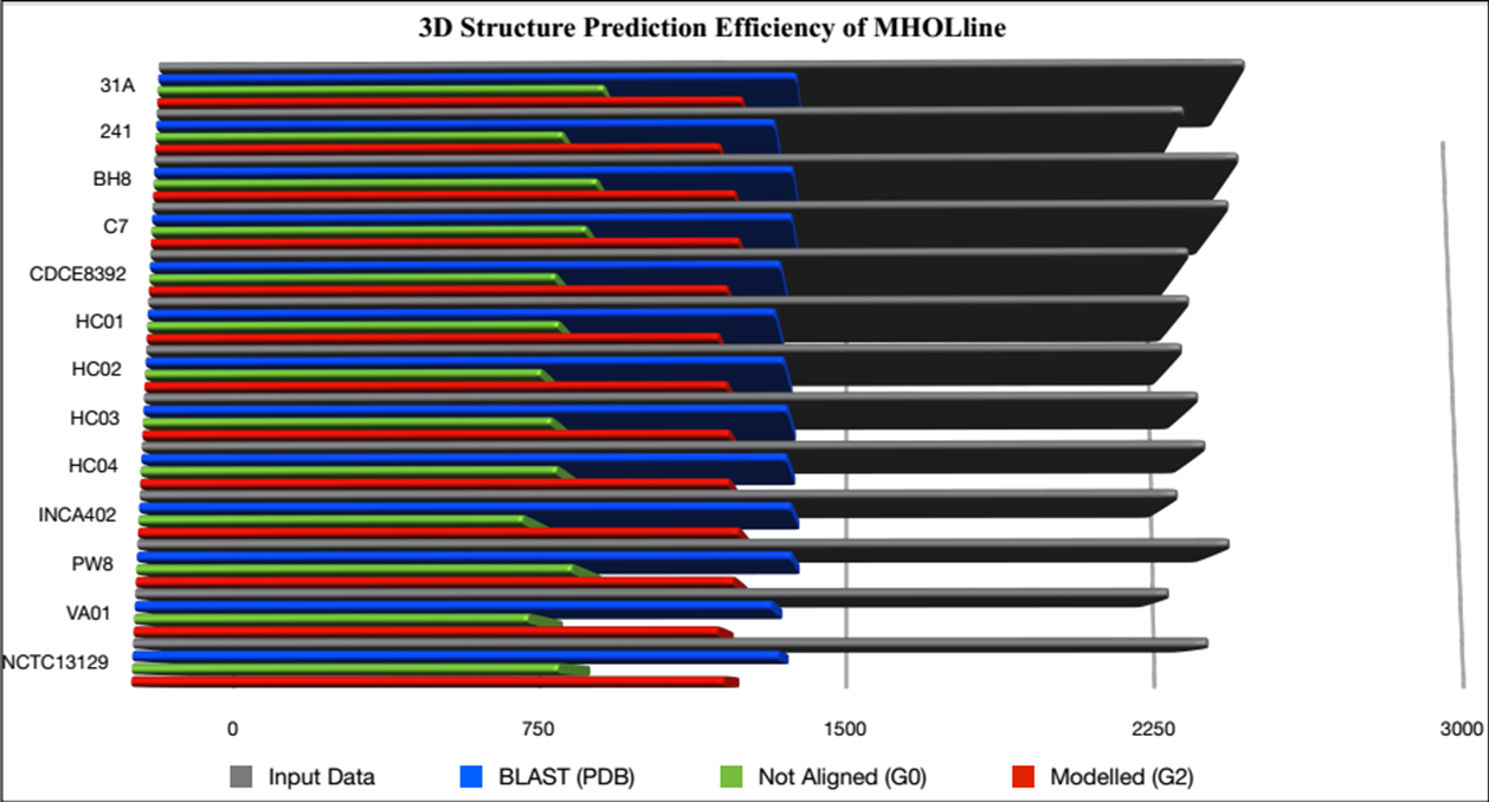
Efficiency of the MHOLline biological workflow for genome-scale modelome (3D models) prediction. Predicted proteomes from the genomes of 13 *C*. *diphtheriae* strains were fed to the MHOLline workflow in FASTA format. The grey bars represent the number of input data. The remaining bars (MHOLline output data) show the number of not aligned sequences (G0, green bars), sequences for which there is a template structure available at RCSB PDB (blue bars), and sequences with acceptable template structures that were modeled in the MHOLline workflow (G2, red bars).

Among 13 strains of *C*. *diphtheriae* species, our employed methodology produced high-confidence 3D structural models from orthologous proteins in *C*. *diphtheriae* species through the efficient MHOLline workflow (**Fig 3**). A comparative structural genomics approach was followed where all the G2 sequences classified as “Very High”, “High”, “Good” and “Medium to Good quality” by MHOLline, from the 12 Cd strains, were aligned to the Cd NCTC13129 strain as a reference genome. First, we identified a set of common conserved proteins with a pre-defined sequence similarity of 95–100%. This resulted in a set of 463 protein sequences, being conserved in all Cd strains (**S3 Table**).

### Protein targets as putative drug and vaccine candidates

The identification of essential proteins in *C*. *diphtheriae* was carried out where the core-modelome was compared to DEG (Database of Essential Genes). This filter drastically reduced the number of selected targets to 23 final targets. Further comparison of the corresponding protein sequences to the human host proteome resulted in a set of 8 targets as essential non-host homologous (ENH, **Table 2**) and a set of 15 targets as essential host homologous proteins (EH, **Table 3**).

**Table 2:** Drug and/or vaccine target prioritization parameters and functional annotation of the eight essential non-host homologous putative targets.

Gene and protein codes	Official full name	Cavities with DS^a^ > 0.80	Cavities with DS^a^^,^^g^ > 0.60 and < 0.80	Mol. Wt^b^ (KDa)	Functions^c^	Cellular component^d^	Pathways^e^	Virulence^f^
NP_939692.1, **nusB**	Transcription antitermination protein NusB/ N utilization substance protein B homolog	0	2	20.382	**MF:** RNA binding. **BP:** DNA-templated transcription, termination, regulation of transcription, DNA-templated.	Cytoplasm	unknown	No
NP_939612.1, **hisE**	Phosphoribosyl-ATP pyrophosphatase	0	1	9.877	**MF:** RNA binding, phosphoribosyl-ATP diphosphatase activity **BP:** histidine biosynthetic process	Cytoplasm	Biosynthesis of amino acids	Yes
NP_939445.1, **DIP1084**	Iron ABC transporter membrane protein/ Putative iron transport membrane protein, FecCD-family	2	3	35.470	**MF:** Transporter activity **BP:** Transport	Membrane	The ATP-binding cassette (ABC) transporters form one of the largest known protein families	Yes
NP_939345.1, **DIP0983**	Hypothetical protein DIP0983/ Uncharacterized protein	0	4	28.193	**MF:** possible lysine decarboxylases (Pfam)/52% sequence identity with PDB Template 1WEK. **BP:** A pyridoxal-phosphate protein. Also acts on 5-hydroxy-L-lysine (IUBMB Comments)	Cytoplasm	unknown	Yes
NP_939302.1, **glpX**	Fructose 1,6-bisphosphatase II	3	2	35.589	**MF:** fructose 1,6-bisphosphate 1-phosphatase activity, metal ion binding. **BP:** gluconeogenesis, glycerol metabolic process		Carbohydrate Metabolism	No
NP_939123.1, **smpB**	SsrA-binding protein	1	2	18.784	**MF:** RNA binding	Cytoplasm	unknown	Yes
NP_938900.1, **rpsH**	30S ribosomal protein S8	1	1	14.292	**MF:** rRNA binding, structural constituent of ribosome **BP:** Translation	Extracellula/ Cytoplasm	unknown	No
NP_938502.1, **bioB**	Biotin synthase	3	1	38.224	**MF:** 2 iron, 2 sulfur cluster binding, 4 iron 4 sulfur cluster binding, biotin synthase activity, iron ion binding **BP:** biotin biosynthetic process	Cytoplasm	Biotin metabolism	Yes

^a^Druggability predicted with DoGSiteScorer software. A druggability score above 0.60 is considered to be good, but a score above 0.80 is favored [32].

^b^Molecular weight was determined using ProtParam tool (http://web.expasy.org/protparam/).

^c^Molecular function (MF) and biological process (BP) for each target protein was determined using UniProt.

^d^Cellular localization of pathogen targets was performed using CELLO.

^e^KEGG was used to find the role of these targets in different cellular pathways.

^f^PAIDB was used to check if the putative targets are involved in the pathogen’s virulence.

^g^DS = Drug Score

**Table 3:** Drug and/or vaccine target prioritization parameters and functional annotation of the fifteen essential host homologous putative targets.

Gene and protein codes	Official full name	Cavities with DS^a^ > 0.80	Cavities with DS^a^^,^^g^ > 0.60 and < 0.80	Mol. Wt^b^. (KDa)	Functions^c^	Cellular^d^ component	Pathways^e^	Virulence^f^
NP_938651.1 **RecR**	Recombination protein RecR	0	2	**23.901**	**MF:** DNA binding, metal ion binding **BP:** DNA recombination, DNA repair	Cytoplasm	Homologous recombination	**Yes**
NP_938792.1 **DIP0411**	Putative electron transport related protein	**0**	2	**19.950**	**MF:** Antioxidant activity, oxidoreductase activity	Cytoplasm/ Membrane	**—**	**Yes**
NP_938922.1 **rpsM**	30S ribosomal protein S13	**0**	1	**13.777**	**MF:** rRNA binding, structural constituent of ribosome, RNA binding **BP:** Translation	Cytoplasm	Ribosome	**No**
NP_939046.1 **DIP0672**	Putative uptake hydrogenase small subunit	**2**	0	**43.949**	**MF:** 3 iron, 4 sulfur cluster binding, 4 iron, 4 sulfur cluster binding, ferredoxin hydrogenase activity, metal ion binding	Cytoplasm	Microbial metabolism in diverse environments	**Yes**
NP_939341.1 **dapD**, DIP0979	Tetrahydropicolinate succinylase **EC 2.3.1.117**	**1**	1	**33.780**	**MF:** 2,3,4,5-tetrahydropyridine-2,6-dicarboxylate N-succinyltransferase activity, magnesium ion binding **BP:** diaminopimelate biosynthetic process, lysine biosynthetic process via diaminopimelate	Cytoplasm	Biosynthesis of amino acids	**Yes**
NP_939343.1 **DIP0981**	Putative succinyltransferase **EC 2.3.1.117**	**1**	1	**33.039**	**MF:** 2,3,4,5-tetrahydropyridine-2,6-dicarboxylate N-succinyltransferase activity	Cytoplasm	Biosynthesis of amino acids	**Yes**
NP_939460.1 **ilvH**, DIP1099	Acetolactate synthase small subunit **EC 2.2.1.6**	**1**	3	**19.063**	**MF:** acetolactate synthase activity, amino acid binding **BP:** branched-chain amino acid biosynthetic process	Cytoplasm/ Membrane	2-Oxocarboxylic acid metabolism	**Yes**
NP_939590.1 **cobM**	Precorrin-4 C11-methyltransferase **EC 2.1.1.133**	**1**	2	**27.181**	**MF:** precorrin-2 dehydrogenase activity, precorrin-4 C11-methyltransferase activity **BP:** cobalamin biosynthetic process, porphyrin-containing compound biosynthetic process	Cytoplasm	Porphyrin and chlorophyll metabolism	**Yes**
NP_939786.1 **DIP1438**	Putative transport membrane protein	**4**	3	**44.215**	**MF: T**ransporter activity **BP:** transmembrane transport	Membrane	The ATP-binding cassette (ABC) transporters	**Yes**
NP_939832.1 **DIP1484**	Putative uroporphyrinogen III methyltransferase	**3**	2	**28.296**	**MF:** Methyltransferase activity **BP:** oxidation-reduction process	Cytoplasm	Porphyrin and chlorophyll metabolism	**Yes**
NP_939958.1 **aroH,** DIP1616	Phospho-2-dehydro-3-deoxyheptonate aldolase **EC 2.5.1.54**	**2**	3	**50.805**	**MF:** 3-deoxy-7-phosphoheptulonate synthase activity **BP:** aromatic amino acid family biosynthetic process	Cytoplasm	Biosynthesis of amino acids	**Yes**
NP_940018.1 **DIP1680**	Putative GTP cyclohydrolase 1 type 2 **EC 3.5.4.16**	**2**	1	**40.657**	**MF:** GTP binding, GTP cyclohydrolase I activity, metal ion binding **BP:** 7,8-dihydroneopterin 3'-triphosphate biosynthetic process	Cytoplasm	—	**Yes**
NP_940228.1 **cysE,** DIP1891	Serine acetyltransferase **EC 2.3.1.30**	**1**	0	**20.208**	**MF:** serine O-acetyltransferase activity **BP:** cysteine biosynthetic process from serine	Cytoplasm	Carbon metabolism	**Yes**
NP_940284.1 **DIP1952**	Putative pyruvate dehydrogenase	**3**	1	**62.497**	**MF:** Catalytic activity, magnesium ion binding, thiamine pyrophosphate binding	Cytoplasm	(PYRUVATE METABOLISM) Nicotinate and nicotinamide metabolism	**Yes**
NP_940605.1 **DIP2303**	Putative DNA protection during starvation protein	**0**	1	**18.223**	**MF:** Ferric iron binding, oxidoreductase activity, oxidizing metal ions **BP:** cellular iron ion homeostasis, response to stress	Cytoplasm	—	**Yes**

^a^Druggability predicted with DoGSiteScorer software. A druggability score above 0.60 is usually considered, but a score above 0.80 is favored [32].

^b^ Molecular weight was determined using ProtParam tool (http://web.expasy.org/protparam/).

^c^ Molecular function (MF) and biological process (BP) for each target protein was determined using UniProt.

^d^Cellular localization of pathogen targets was performed using CELLO.

^e^KEGG was used to find the role of these targets in different cellular pathways.

^f^PAIDB was used to check if the putative targets are involved in the pathogen’s virulence.

^g^DS = Drug Score.

### Prioritization parameters for drug targets and vaccine candidates

There are several factors that can aid in determining potential therapeutic targets [30]. For vaccine candidates, the information about subcellular localization is important: Proteins that contain transmembrane motifs are favored [24, 30, 38, 39]. The 23 essential proteins have a low molecular weight and all are localized in the cytoplasmic compartment of *C*. *diphtheriae* (**Tables 2 & 3**). After the druggability evaluation using DoGSiteScorer [32] for both essential non-host and host homologous conserved targets from *C*. *diphtheriae*, we could predict at least one druggable cavity for each Cd target. The host homologous proteins as therapeutic targets could adversely affect the host. Therefore, the first step in numerous *in silico* drug target identification approaches are filtering proteins homologous to host proteome. Thus, we only consider the eight pathogen-essential non host homologs for the docking studies [13, 15, 40]. For the eight pathogen-essential non host homologs (**S2 Table**) glpX, nusB, rpsH, hisE, DIP1084, DIP0983, smpB, and bioB 3, 0, 1, 0, 2, 0, 1 and 3 cavities with score > 0.80 were predicted. The cavity of each protein exhibiting the highest druggability score was subjected to docking analyses. The numbers of predicted cavities with their respective druggability scores are given in **Tables 2 & 3**.

The identified eight non-host homologous and essential Cd proteins could be novel therapeutic targets for *Corynebacterium diphtheriae*.

As per our knowledge, glpX, hisE and bioB proteins have been reported as potential drug target in *Mtb*. Protein nusB is a member of Nus-transcription Factor family that help bacteria in the process of elongation, transcription: translation coupling and termination. Some members of this family (nusG) has already been reported as drug target. Furthermore, rpsH and smpB are also reported as potential drug target by Folador et al., 2016 in their *in silico* study [41]. Protein DIP1084 is Putative iron transport membrane protein (FecCD-family) and DIP0983 is uncharacterized Hypothetical Protein that need to be characterized experimentally. Hence, these protein could be a good therapeutic target against Cd.

### Virtual screening and molecular docking

For each target protein (glpX, nusB, rpsH, hisE, DIP1084, DIP0983, smpB, and bioB) four different libraries were separately screened. A total of 28 molecules from natural compounds library and 31 compounds from the derivatives of Di-terpenoid Iso-steviol library were docked. Furthermore, top 200 drug-like molecules from virtual screening analyses of two large libraries (ZINC drug-like molecules, ZINC Natural Product) were examined one-by-one for the selection of the final set of promising molecules that showed favorable interactions with the ENH targets. The biological importance and an analysis of the predicted protein-ligand interaction/s for each target are described here. The molecule names, ZINC codes and MolDock scores for the selected ligands, as well as the number of predicted hydrogen bonds with the protein cavity residues involved in these interactions, are shown below (**Tables 4–11**) for each target protein. The predicted binding modes of selected ligands are also shown for each pathogen target in **Figs 5–12.**

### Validation of docking protocol

**Fig 4:**
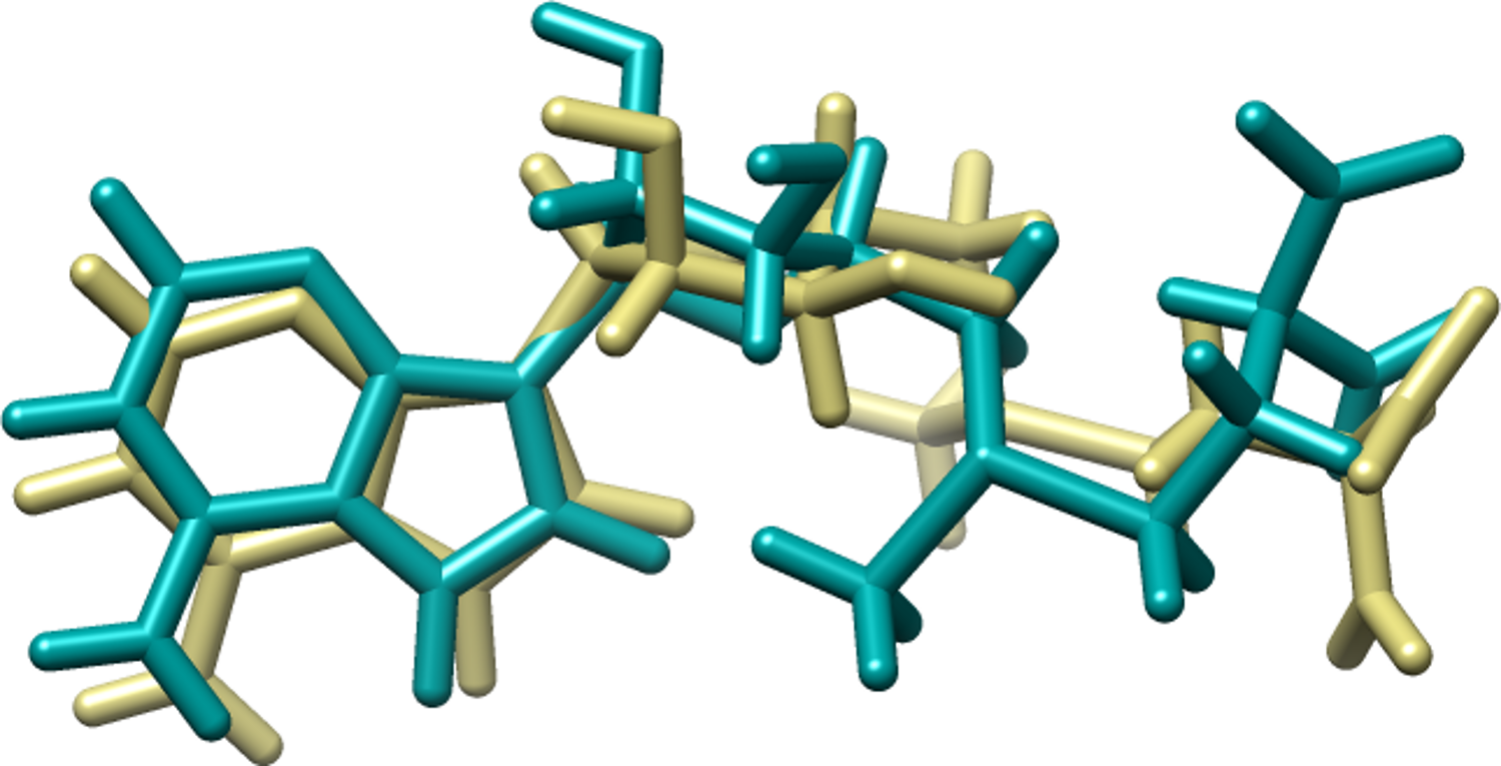
Superposition of co-crystallized and Docked ligand; Dark Khaki represents the co crystallized ligand and Dark Cyan the re-docked conformation of the ligand.

To validate the accuracy of MolDock program (MVD), the co-crystallized ligand of Biotin synthase, bioB (PDB ID; 1R30) was extracted and then re-docked into the binding pocket of receptor protein. The RMSD between docked and co-crystallized ligand was found to be 1.81 A˚, which shows that the adopted docking protocol is valid and can be used to correctly predict the binding pose of the ligands [35, 42]. The superposition of co-crystallized ligands and docked is shown in **Fig 4.**

**Fig 5:**
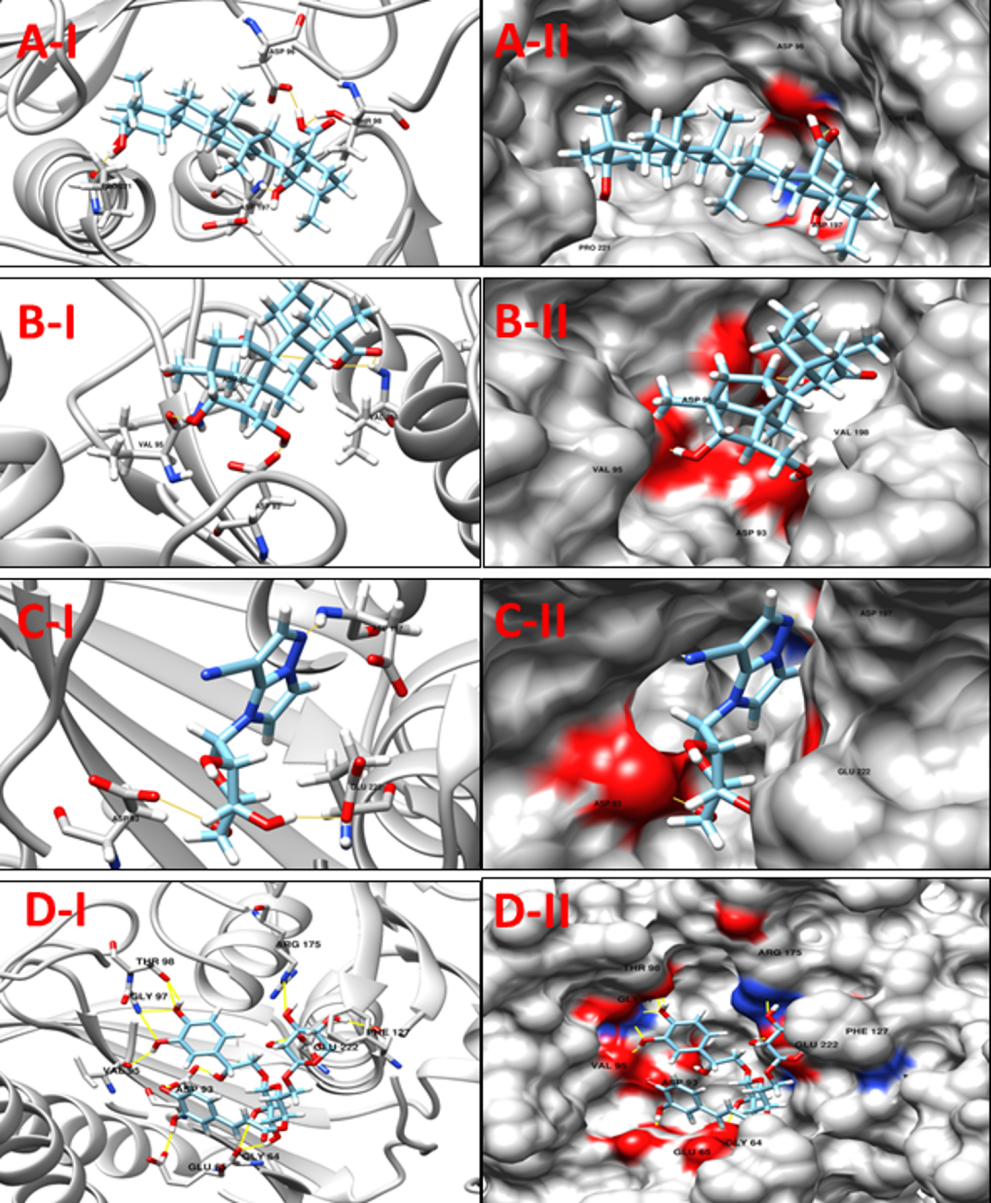
**A-I:** 3D cartoon representation of the docking analyses for the most druggable protein cavity of **NP_939302.1** (glpX, Fructose 1,6-bisphosphatase II) with Jacarandic Acid (CID 73645). **A-II:** 3D surface representation of the docking analyses for the structures of Jacarandic Acid with glpX protein. Figs **B-I, II**, **C-I, II** & **D-I, II** represent same information for compounds 16-hydrazonisosteviol, **ZINC13142972** and **ZINC67912153** respectively, for the same protein cavity.

**NP_939302.1** (**glpX,** Fructose 1, 6-bisphosphatase II) is a key enzyme of gluconeogenesis and catalyzes the hydrolysis of fructose 1, 6-bisphosphate to form fructose 6-phosphate and orthophosphate. A reverse reaction catalyzed by phosphofructokinase in glycolysis, and the product, fructose 6-phosphate, is an important precursor in various biosynthetic pathways [43]. In all organisms, gluconeogenesis is an important metabolic pathway that allows the cells to synthesize glucose from non-carbohydrate precursors, such as organic acids, amino acids and glycerol. FBPases are members of the large superfamily of lithium sensitive phosphatases, which includes three families of inositol phosphatases and FBPases (the phosphoesterase clan CL0171, 3167 sequences, Pfam data base). The FBPases are already reported as targets for the development of drugs for the treatment of noninsulin dependent diabetes [44, 45]. Based on a comparison with a crystallographic structure of the glpX template (PDB ID: 1NI9, GlpX from *Escherichia coli*), none of the active site residues were identified. The docking analysis was performed utilizing the highest scoring pocket obtained from DoGSiteScorer. **Table 4**shows a set of 10 promising ligands according to their minimum energy values and the maximum number of hydrogen bond interactions from the four aforementioned libraries. Compounds **ZINC67912153, ZINC13142972,** Jacarandic Acid and 16-hydrazonisosteviol are shown in **Fig 5**.

**Table 4:** Compounds/Libraries name, MolDock scores and predicted hydrogen bonds for the selected best-ranked molecules against NP_939302.1 (glpX, Fructose 1,6-bisphosphatase II).

Compounds		MolDock Score	H-Bond/Residues
**Plant derived natural compounds**	Rhein	-64.1265	3/ Val95, Asp197
	Jacarandic Acid	-62.0658	4/ Asp96, Thr98, Asp197, Pro221
**Derivative of diterpenoid isosteviol**	16-hydrazonisosteviol	-64.2107	5/ Asp93, Val95, Asp96, Val198
	16-oxime, 17-hydroxyisosteviol	-69.6824	4/ Asp93, Asp96, Thr98
	Benzyl ester isosteviol lactone	-69.8464	3/ Asp93, Asp197, Glu222
**ZINC Compounds**	ZINC00042420	-106.97	3/ Arg175, Arg197, Val198
	ZINC13142972	-109.648	3/ Asp93, Asp197, Glu222
**ZINC** *NP Compounds	ZINC67912153	-135.111	13/Gly64, Glu65, Asp93, Val95, Gly97, Thr98, Phe127, Arg175, Glu222
	ZINC67902753	-121.762	8/Glu65, Val95, Thr98, Glu222
	ZINC38143633	-123.150	11/Lys37, Asp93, Val95, Asp96, Gly97, Thr98

*NP = Natural Product (http://zinc.docking.org/catalogs/acdiscnp)

**Fig 6:**
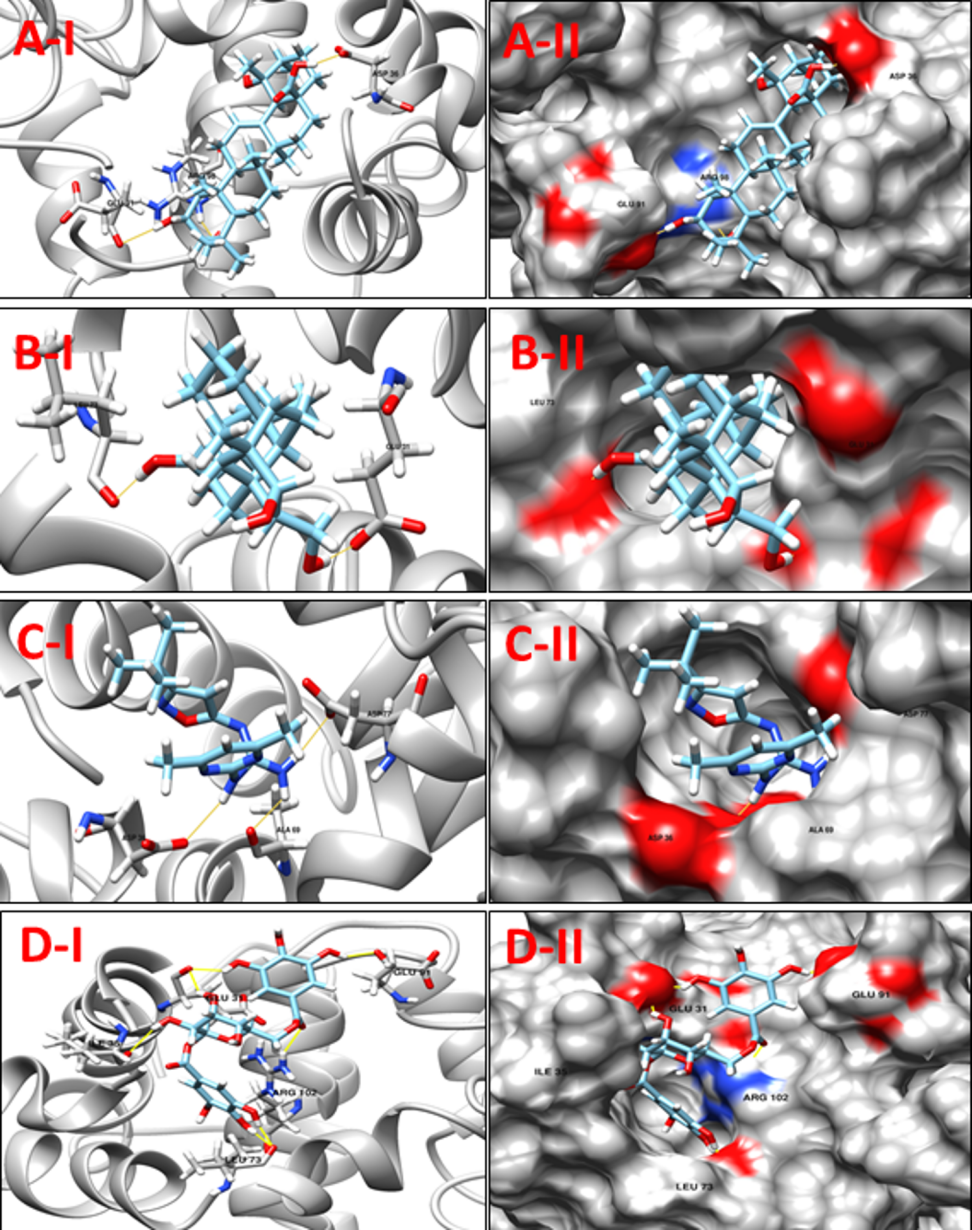
**A-I:** 3D cartoon representation of the docking analyses for the most druggable protein cavity of **NP_939692.1** (**nusB,** Transcription antitermination protein NusB) with Jacarandic Acid (CID 73645). **A-II:** 3D surface representation of the docking analyses for the structures of Jacarandic Acid with nusB protein. Figs **B-I, II**, **C-I, II** and **D-I, II** represent same information for compounds 16-hydrazonisosteviol, **ZINC00053531** and **ZINC15043210** respectively, for the same protein cavity.

**NP_939692.1** (**nusB,** Transcription antitermination protein NusB) is a prokaryotic transcription factor involved in antitermination processes, during which it interacts with the mRNA nut site at boxA portion. The crystal structure of *M*. *tuberculosis* and *E*. *coli* NusB proteins suggest that the basic N-terminal region of the molecule associates with the rRNA BoxA. Hypothetically, this is indicative of the so-called arginine rich RNA binding motif (ARM) in the bacteriophage N protein, HIV tat and HIV rev. This suggestion is supported by the presence of a phosphate-binding site at the N-terminal end of α-A in each NusB protomer that includes a pair of conserved arginines, Arg10 and Arg14 [46]. The bismuth-dithiol solutions have been shown to selectively inhibit *Escherichia coli* rho transcription termination factor [47]. A comparison between the crystallographic structures of the NusB template (PDB ID: 1EYV, NusB from *M*. *tuberculosis*) and our modeled structure reveals that the conserved arginines were located at position 12 and 16 (Arg12 and Arg16) and are likely to contribute in the interactions. Although none of these residues are predicted to form hydrogen bonds with selected docked ligands, these molecules were predicted to interact with other residues in the pocket. **Table 5**shows the 8 selected ligands from all the four libraries according to their minimum energy values and the number of hydrogen bond interactions. The compounds **ZINC15043210, ZINC00053531** Jacarandic Acid and 16-hydrazonisosteviol are shown in **(Fig 6)**. A decent binding mode and good shape complementarity was observed in these complexes.

**Table 5:** Compounds/Libraries name, MolDock scores and predicted hydrogen bonds for the selected molecules against NP_939692.1 (nusB, Transcription antitermination protein NusB).

Compounds		MolDock Score	H-Bond/Residues
**Plant derived natural compounds**	Rhein	-78.0652	1/ Asp36
	Jacarandic Acid	-68.1913	3/ Asp36, Glu91, Arg98
**Derivative of diterpenoid isosteviol**	16-hydrazonisosteviol	-92.7911	2/ Glu31, Leu73
**ZINC Compounds**	ZINC00053531	-99.4716	3/ Asp34, Asp36, Ala69
	ZINC19899354	-114.966	3/ Asp36, Ala69, Asp77
**ZINC NP Compounds**	ZINC67911826	-131.288	7/Ala30, Glu31, Asp34, Ile35, Arg102
	ZINC15043210	-124.580	7/ Glu31, Ile35, Leu73 Glu91, Arg102
	ZINC31168395	-131.442	6/ Glu31, Asp34, Ala69, Leu73, Asp77, Arg102

**Fig 7:**
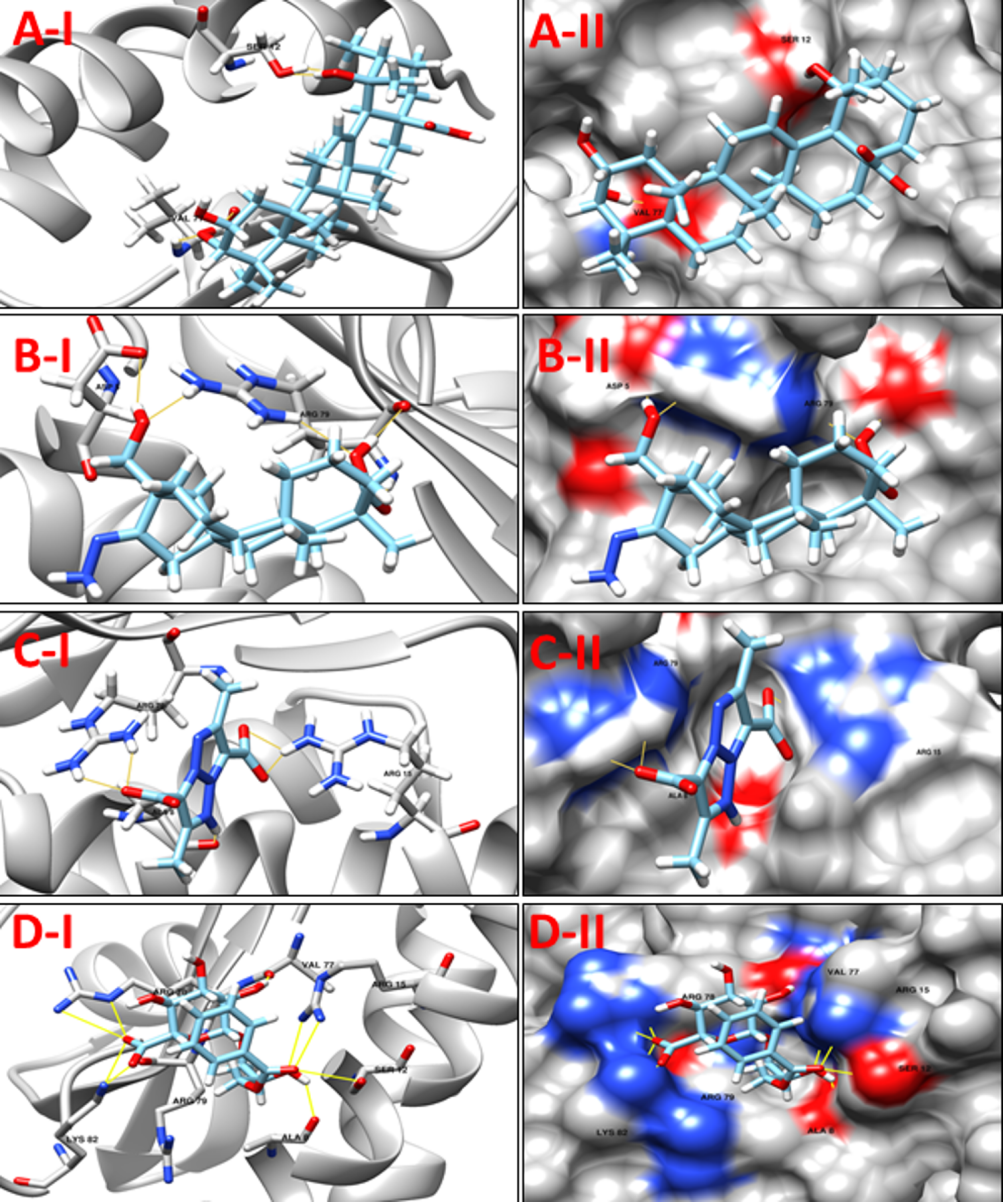
**A-I** 3D cartoon representation of the docking analyses for the most druggable protein cavity of **NP_938900.1** (**rpsH,** 30S ribosomal protein S8) with Jacarandic Acid (CID 73645). **A-II:** 3D surface representation of the docking analyses for the structures of Jacarandic Acid with rpsH protein. Figs **B-I, II**, **C-I, II** and **D-I, II** represent same information for compounds 17-hydroxyisosteviol **ZINC15221730** and **ZINC35457686** respectively, for the same cavity.

**NP_938900.1** (**rpsH,** 30S ribosomal protein S8) is an important RNA-binding protein that inhabits a central position within the small ribosomal subunit. It widely interacts with 16S rRNA and is vital for the correct folding of the central domain of the rRNA. The protein rpsH S8 also controls the synthesis of numerous ribosomal proteins by binding to mRNA. It binds exactly to very similar sites in the two RNA molecules. It is a ribosomal protein that has medium-size, and its role as a significant primary RNA-binding protein in the 30S subunit is discovered recently. The S8 mutations within the protein have been shown to result in defective ribosome assembly. In *Escherichia coli*, the S8-binding site within 16S rRNA has been investigated independently by a number of techniques including nuclease protection, RNA–protein crosslinking, RNA modification, hydroxyl-radical footprinting and chemical probing. The rpsH S8 protein is also one of the principal regulatory elements that control ribosomal protein synthesis by the translational feedback inhibition mechanism discovered by Nomura and colleagues [48]. It regulates the expression of the spc operon that encodes, in order, the ten ribosomal proteins L14, L24, L5, S14, S8, L6, L18, S5, L30 and L15 [49]. The active site residues of rpsH, based on a comparison with its template structure were Arg86, Tyr88, Ser107, Ser109, Gly124, Gly125 and Glu126. However, none of the molecules interacts with these residues (**Table 6**); nonetheless they are predicted to interact with other residues of the binding cavity predicted by DoGSiteScorer. The predicted binding mode of best scoring compounds each library **ZINC35457686**, **ZINC15221730**, Jacarandic Acid and 17-hydroxyisosteviol are shown in **Fig 7.**

**Table 6:** Compounds/Libraries name, MolDock scores and predicted hydrogen bonds for the selected molecules against NP_938900.1 (rpsH, 30S ribosomal protein S8).

Compounds		MolDock Score	H-Bond/Residues
**Plant derived natural compounds**	Rhein	-48.8803	3/ Asp5, Arg15
	Jacarandic Acid	-49.3506	4/ Ser12, Val77
**Derivative of diterpenoid isosteviol**	16-hydrazonisosteviol	-68.2446	3/ Asp5, Arg13, Arg79
	17-hydroxyisosteviol	-64.5855	5/ Asp5, Arg79
	16–17 dihydroxyisosteviol	-56.3868	4/ Asp5, Arg79
	16-oxime, 17-hydroxyisosteviol	-65.1995	4/ Ser26, Ser29, Ser30
**ZINC Compounds**	ZINC15221730	-103.636	5/ Ala8, Arg15, Arg79
	ZINC71913776	-87.1474	5/ Arg15, Arg79
	ZINC72333100	-104.807	3/ Arg15, Arg79, Val80
**ZINC NP Compounds**	ZINC35457686	-107.091	10/Ala8, Ser12, Arg15, Val77, Arg78, Arg79, Lys82
	ZINC67903079	-131.210	10/ Asp5, Ser12, Arg15, Arg78, Arg79, Lys82
	ZINC31163223	-100.684	7/ Ala8, Asp9, Arg78, Arg79, Lys82

**Fig 8:**
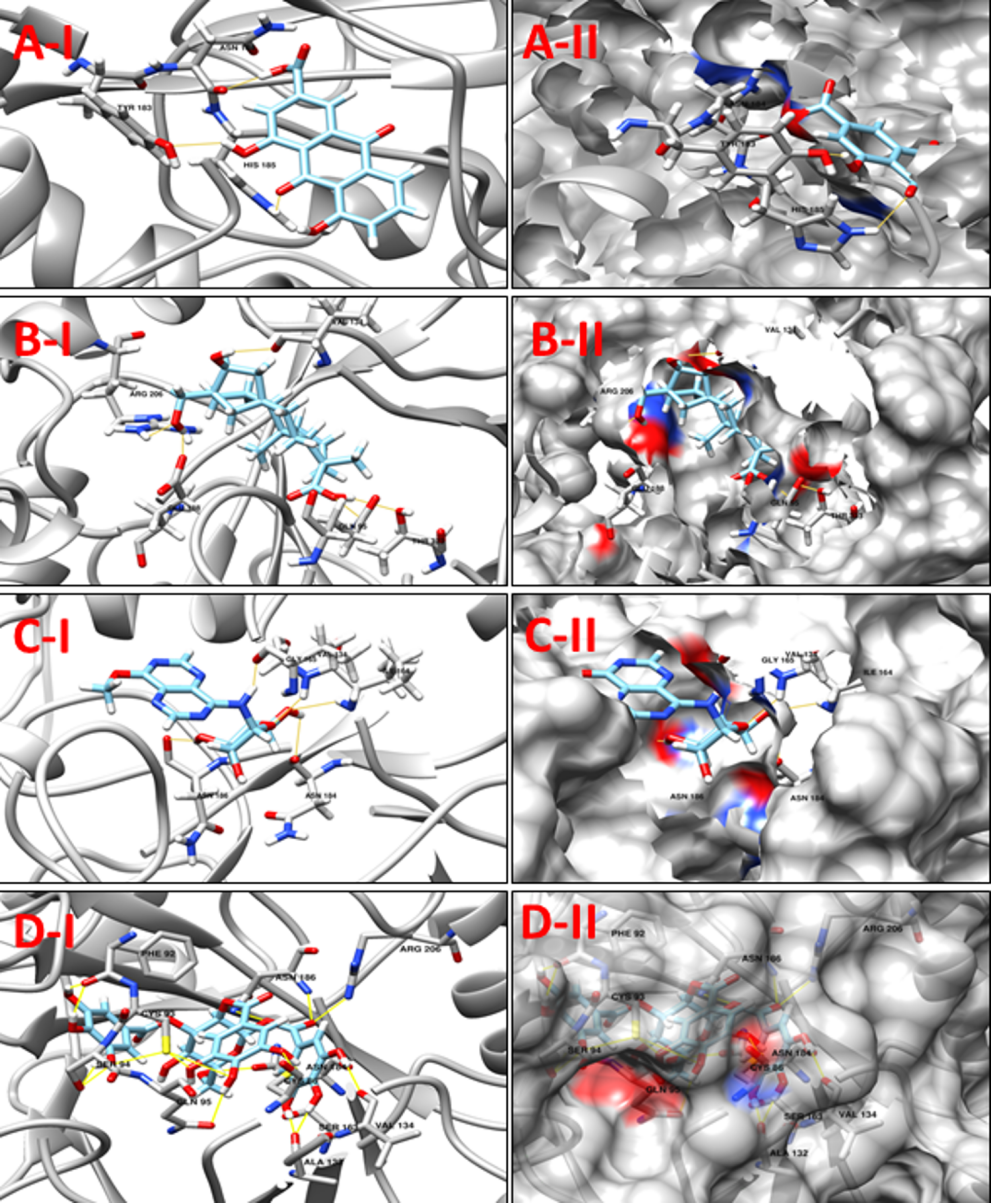
**A-I** 3D cartoon representation of the docking analyses for the most druggable protein cavity of **NP_938502.1** (**bioB,** Biotin synthase) with Rhein (CID 10168). **A-II:** 3D surface representation of the docking analyses for the structure of Rhein with bioB protein. Figs **B-I, II**, **C-I, II** & **D-I, II** represent same information for compounds 16-oxime, 17-hydroxyisosteviol, **ZINC16952914** and **ZINC77269615** respectively, for the same protein cavity.

**NP_938502.1** (**bioB,** Biotin synthase) catalyzes the final step in the biotin biosynthetic pathway by converting dethiobiotin (DTB) to biotin. This reaction uses organic radical chemistry for inserting sulfur atom between non activated carbons C6 and C9 of DTB. BioB is a member of the “radical SAM” or “AdoMet radical” superfamily, which is categorized by the presence of a conserved CxxxCxxC sequence motif (C, Cys; x, any amino acid) that synchronizes an essential Fe_4_S_4_ cluster, as well as by the use of S-adenosyl-Lmethionine (SAM or AdoMet) for radical generation. AdoMet radical enzymes act on a wide variety of biomolecules. For example, BioB and lipoyl-acyl carrier protein synthase (LipA) are involved in vitamin biosynthesis; lysine 2,3-aminomutase (LAM) facilitates the fermentation of lysine; class III ribonucleotide reductase (RNR) and pyruvate formate lyase (PFL) catalyze the formation of glycyl radicals in their respective target proteins; and spore photoproduct lyase repairs ultraviolet light-induced DNA damage [50]. The protein bioB was reported as putative drug target in *C*. *diphtheriae* by Barh *et al*., 2011 in their in silico study [15]. A comparison between our modeled protein and template structures suggest Cys86, Cys90, Cys93 and Arg291 as the active residues. Although, only Cys86, Cys90 and Cys93 were found to interact with the compounds from our prepared libraries, the molecules were predicted to interact with other residues in the pocket. The binding mode of compounds with active site residues and low scores suggest a set of 10 molecules (**Table 7**) as promising leads from our four libraries. The predicted binding modes of Jacarandic Acid, 16-oxime, 17-hydroxyisosteviol, **ZINC16952914** and **ZINC77269615** are shown in **Fig 8**.

**Table 7:** Compounds/Libraries name, MolDock scores and predicted hydrogen bonds for the selected molecules against NP_938502.1 (bioB, Biotin synthase).

Compounds		MolDock Score	H-Bond/Residues
**Plant derived natural compounds**	Rhein	-72.2918	3/ Tyr183, Asn184, His185
	Jacarandic Acid	-98.0169	2/ Ala132, Glu188
**Derivative of diterpenoid isosteviol**	16-hydrazonisosteviol	-107.55	4/ Gly165, Tyr183, Asn184, Glu188
	17-hydroxyisosteviol	-92.2141	4/ Cys93, Ala132, Val134, Tyr183
	16-oxime, 17-hydroxyisosteviol	-98.9592	5/ Glu95, Val134, Glu188, Arg206, Thr323
	Benzyl ester isosteviol lactone	-89.8881	5/ Ala132, Val134, Gly165, Asn184
**ZINC Compounds**	ZINC16952914	-119.354	5/ Val134, Ile164, Gly165, Asn184, Asn186
**ZINC NP Compounds**	ZINC77269615	-164.853	17/Cys86, Phe92, Cys93, Ser94, Gln95, Ala132, Val134, Ser163, Asn184, Asn186, Arg206
	ZINC04098512	-162.050	14/ Cys86, Cys90, Cys93, Ala132, Val134, Asn184, Asn186, His201, Arg206, Asp256, Thr323
	ZINC15112225	-142.080	10/ Phe92, Cys93, Ser94, Gln95, Asn186, Arg206, Asn253

**Fig 9:**
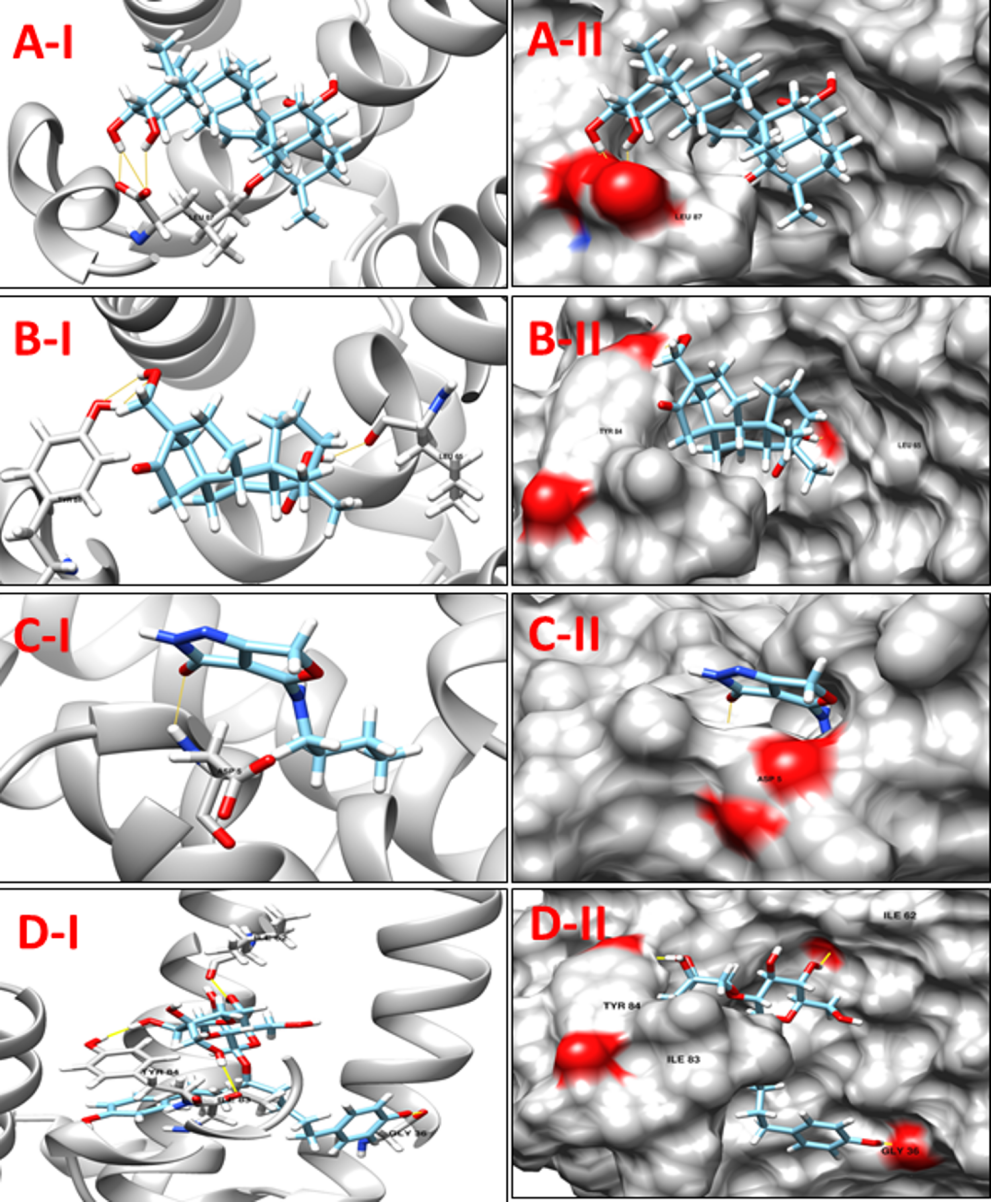
**A-1** 3D cartoon representation of the docking analyses for the most druggable protein cavity of **NP_939612.1** (**hisE,** Phosphoribosyl-ATP pyrophosphatase) with Jacarandic Acid (CID 73645). **A-II:** 3D surface representation of the docking analyses for the structure of Jacarandic Acid with hisE protein. Figs **B-I, II**, **C-I, II** & **D-I, II** represent same information for compounds 16–17 dihydroxyisosteviol, **ZINC05809437** and **ZINC67913372** respectively, for the same protein cavity.

**NP_939612.1** (**hisE,** Phosphoribosyl-ATP pyrophosphatase) is the second enzyme in the histidine-biosynthetic pathway, hydrolyzing irreversibly phosphoribosyl-ATP to phosphoribosyl-AMP and pyrophosphate. It is encoded by the *hisE* gene, which is present as a separate gene in many bacteria and archaea but is fused to *hisI* in other bacteria, fungi and plants. As it is essential for growth as seen in *in vitro* experiments, HisE is a potential drug target for tuberculosis [51]. A comparison of template and target protein structures here showed that there was no reported information about ligand-residue/s association in the active site cavity. Hence, the cavity chosen for virtual screening was simply the one that presented the highest DogSiteScorer druggability score (>80). A list of best dock molecules is shown below (**Table 8**). The binding patterns of Jacarandic Acid, 16–17 dihydroxyisosteviol, **ZINC05809437** and **ZINC67913372** are shown in **Fig 9.**

**Table 8:** Compounds/Libraries name, MolDock scores and predicted hydrogen bonds for the selected molecules against NP_939612.1 (hisE, Phosphoribosyl-ATP pyrophosphatase).

Compounds		MolDock Score	H-Bond/Residues
**Plant derived natural compounds**	Rhein	-54.9556	1/ Tyr84
	Jacarandic Acid	-61.0241	3/ Leu87
**Derivative of diterpenoid isosteviol**	16–17 dihydroxyisosteviol	-70.8496	3/ Leu65, Tyr84
**ZINC Compounds**	ZINC05809437	-89.6781	1/ Asp5
**ZINC NP Compounds**	ZINC38143703	-99.499	4/ Thr79, Ile83, Leu87
	ZINC67913372	-97.997	5/ Gly36, Ile62, Ile83, Tyr84

**Fig 10:**
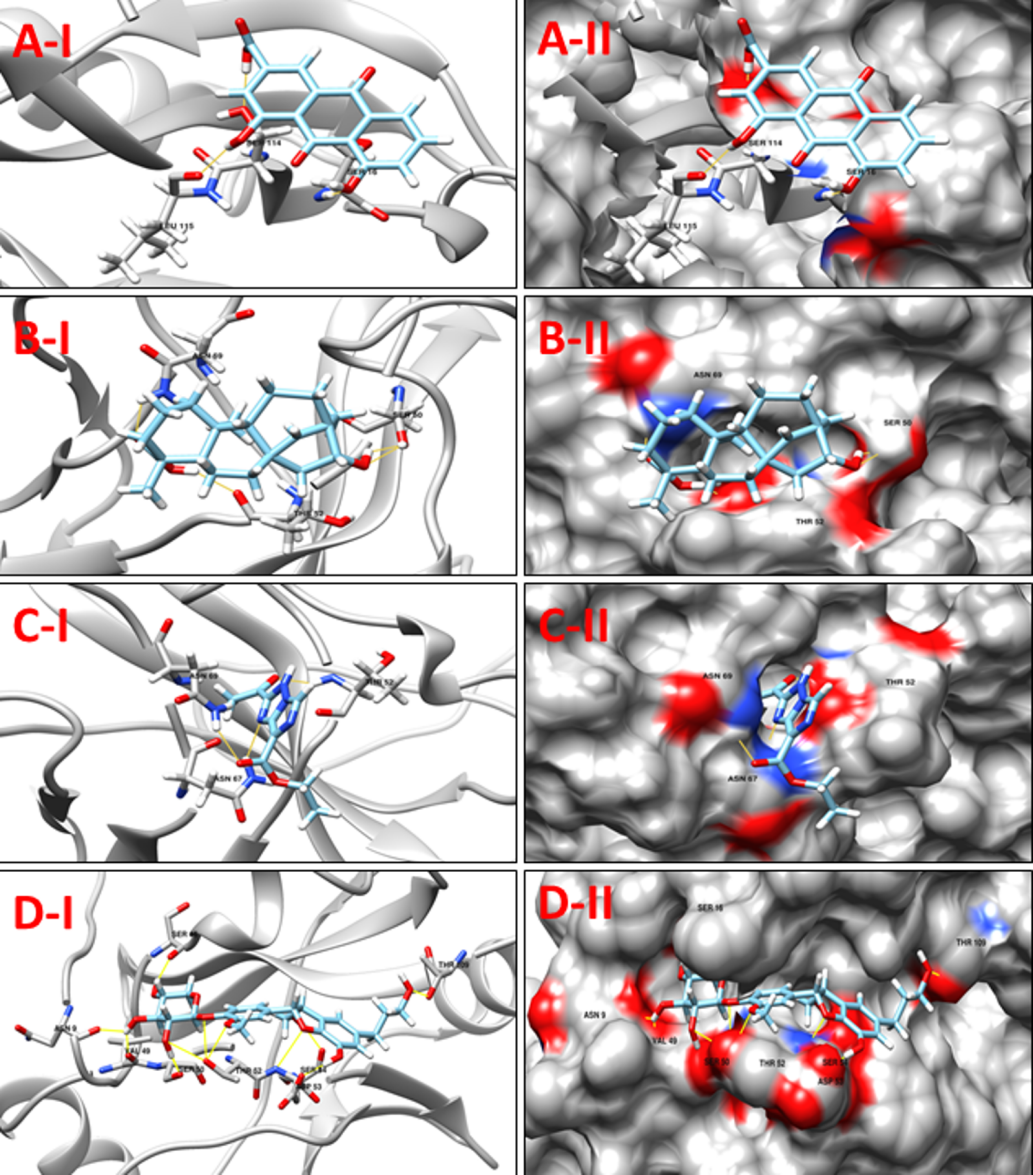
**A-I** 3D cartoon representation of the docking analyses for the most druggable protein cavity of **NP_939123.1** (**smpB,** SsrA-binding protein) with Rhein (CID 10168). **A-II:** 3D surface representation of the docking analyses for the structure of Rhein with smpB protein. Figs **B-I, II**, **C-I, II** & **D-I, II** represent same information for compounds 16-hydroxyisosteviol **ZINC01414475** & **ZINC31168211** respectively, for the same protein cavity.

**NP_939123.1** (**smpB,** SsrA-binding protein) is a small protein B (SmpB), which is very useful for biological functions of tmRNA. In bacteria, a hybrid RNA molecule that combines the functions of both messenger and transfer RNAs rescues stalled ribosomes, and targets aberrant, partially synthesized proteins for proteolytic degradation. The flexible RNA molecule adopts an open L-shaped conformation and SmpB binds to its elbow region, stabilizing the single-stranded D-loop in an extended conformation. The most prominent feature of the structure of tmRNA_**Δ**_ is a 90^o^ rotation of the TѰC-arm around the helical axis. Because of this important conformation, the SmpB–tmRNA D-complex positioned into the A-site of the ribosome orients SmpB towards the small ribosomal subunit, and directs tmRNA towards the elongation-factor binding region of the ribosome. The tmRNA–SmpB rescue system is ubiquitous in bacteria, and is also found in some chloroplasts and mitochondria [52]. In this case the template structure (PDB ID: 1P6V) did not contain any ligand, and no reported information was found about the ligand-residue interaction in their cavities. Therefore, amongst the cavities identified by MVD, the best cavity for docking analysis was chosen in consensus with highest druggability score from the DogSiteScorer. **ZINC31168211** was found to form the network of 12 hydrogen bonds with Asn9, Ser16, Val49, Ser50, Thr52, Asp53, Ser54, Thr109. **Table 9**lists top compounds from respective libraries selected for this target while the binding modes of Rhein, 16-hydroxyisosteviol, **ZINC01414475 and ZINC31168211** are also shown (**Fig 10).**

**Table 9:** Compounds/Libraries name, MolDock scores and predicted hydrogen bonds for the selected molecules against NP_939123.1 (smpB, SsrA-binding protein).

Compounds		MolDock Score	H-Bond/Residues
**Plant derived natural compounds**	Rhein	-67.698	3/ Ser16, Ser114, Leu115
	Jacarandic Acid	-52.3689	1/ Asn69
**Derivative of diterpenoid isosteviol**	16-hydroxyisosteviol	-53.2141	4/ Ser50, Thr52, Asn69
	16-hydrazonisosteviol	-64.6203	3/ Thr52, Asn67
	16–17 dihydroxyisosteviol	-59.7364	3/ Ser16, Lys19, Val49
**ZINC Compounds**	ZINC01414475	-86.7944	3/ Thr52, Asn67, Asn69
	ZINC17128469	-74.5349	3/ Ser16, Leu51, Thr52
**ZINC NP Compounds**	ZINC31168211	-158.056	12/ Asn9, Ser16, Val49, Ser50, Thr52, Asp53, Ser54, Thr109
	ZINC33832449	-134.974	10/ Asn9, Ser16, Asn17, Val49, Ser50, Thr52, Asp53
	ZINC04096316	-137.613	9/ Asn9, Ser10, Ser16, Asn17, Lys19, Val49, Ser50, Thr52

**Fig 11:**
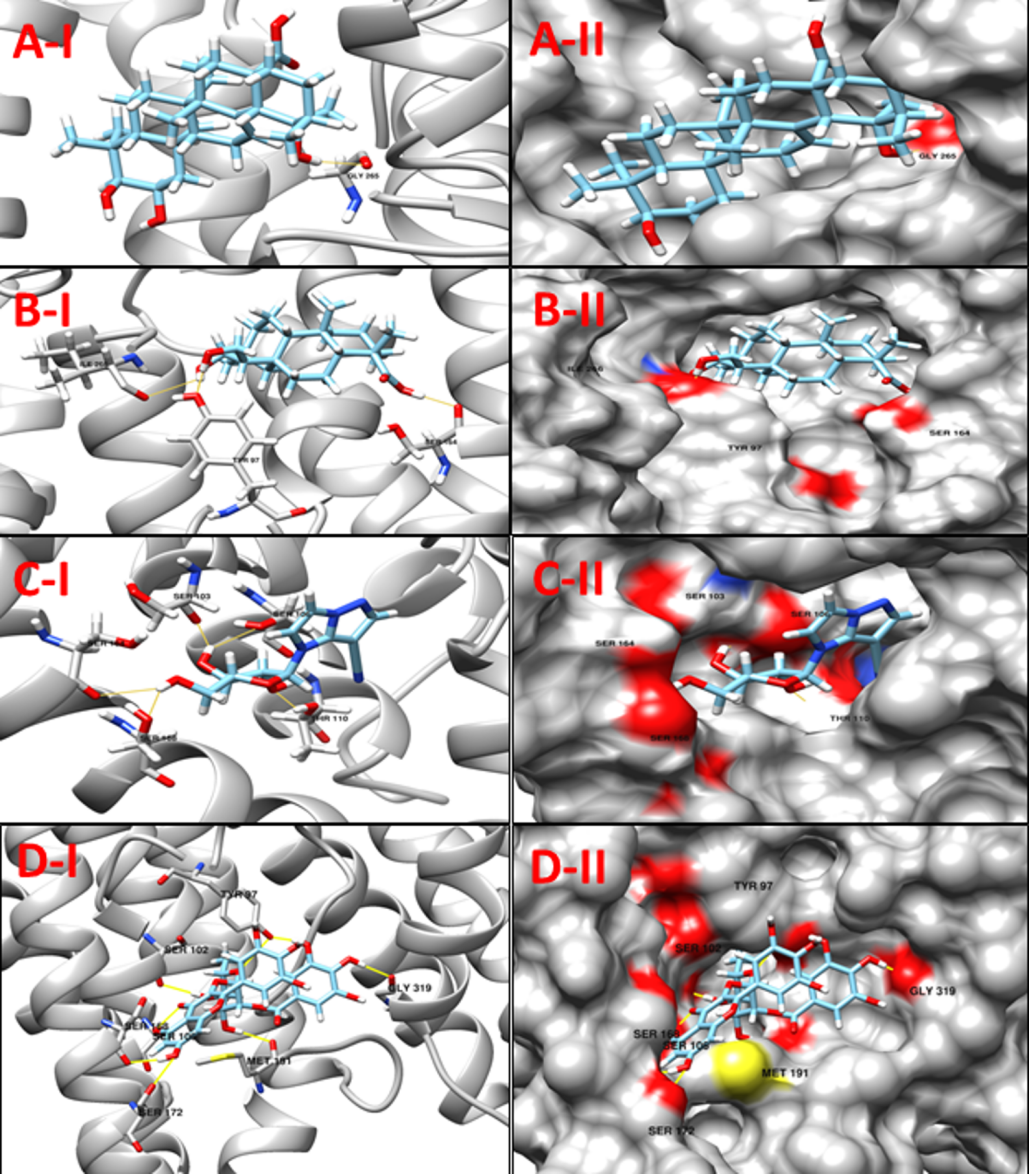
**A-I** 3D cartoon representation of the docking analyses for the most druggable protein cavity of **NP_939445.1** (**DIP1084,** Putative iron transport membrane protein, FecCD-family) with Jacarandic Acid (CID 73645). **A-II:** 3D surface representation of the docking analyses for the structure of Jacarandic Acid with **DIP1084,** Putative iron transport membrane protein. Figs **B-I, II, C-I, II** & **D-1, II D** represent same information for compounds 16-hydrazonisosteviol **ZINC13142972** and **ZINC70454922** respectively, for the same protein cavity.

**NP_939445.1** (**DIP1084,** Putative iron transport membrane protein, FecCD-family) The Pfam search for the protein showed that it has two main components, FecCD and ABC_trans. The FecCD is a subfamily of bacterial binding-protein-dependent transport systems family constituting transport system permease proteins involved in the transport of numerous compounds through the membrane. These transporters tend to catalyze the thermodynamically unfavorable translocation of substrates against a transmembrane concentration gradient through the coupling to a second, energetically favorable process. ABC systems can be categorized in three functional groups, as follows. Importers mediate the uptake of nutrients in prokaryotes. The nature of the substrates that are transported is very wide, including mono- and oligosaccharides, organic and inorganic ions, amino acids, peptides, iron-siderophores, metals, polyamine cations, opines, and vitamins [53]. Exporters are involved in the secretion of various molecules, such as peptides, lipids, hydrophobic drugs, polysaccharides, and proteins, including toxins such as hemolysin. The third category of systems is apparently not involved in transport, with some members being involved in translation of mRNA and in DNA repair. **Table 10**shows a set of 11 high scoring compounds against the proposed target. Compound **ZINC70454922** from ZINC NP library was predicted to form ten hydrogen bonds with relatively low docking score (**Fig 11**).

**Table 10:** Compounds/Libraries name, MolDock scores and predicted hydrogen bonds for the selected molecules against NP_939445.1 (DIP1084, Putative iron transport membrane protein, FecCD-family).

Compounds		MolDock Score	H-Bond/Residues
**Plant derived natural compounds**	Rhein	-66.4406	1/ Ser164
	Jacarandic Acid	-77.5981	1/ Gly265
**Derivative of diterpenoid isosteviol**	16-hydrazonisosteviol	-96.3945	4/ Tyr97, Ser164, Ile266
	17-hydroxyisosteviol	-90.1488	4/ Tyr97, Ser164, Ile266
	Benzyl ester isosteviol lactone	-71.4733	4/ Tyr97, Ser164
**ZINC Compounds**	ZINC01645563	-95.7116	5/ Tyr97, Ser102, Ser164, Ile266
	ZINC13142972	-111.185	5/ Ser103, Ser106, Thr110, Ser164, Ser168
	ZINC62023045	-103.542	4/ Tyr97, Ser102, Ser106, Ser164
**ZINC NP Compounds**	ZINC70454922	-155.667	10/ Tyr97, Ser102, Ser106, Ser168, Ser172, Met191, Gly319
	ZINC31167925	-135.535	10/ Tyr97, Ser164, Ser168, Met191, Gly265, Ile266, Thr322
	ZINC04963990	-127.671	8/ Asp95, Tyr97, Ser106, Met191, Ile266, Phe268

**Fig 12:**
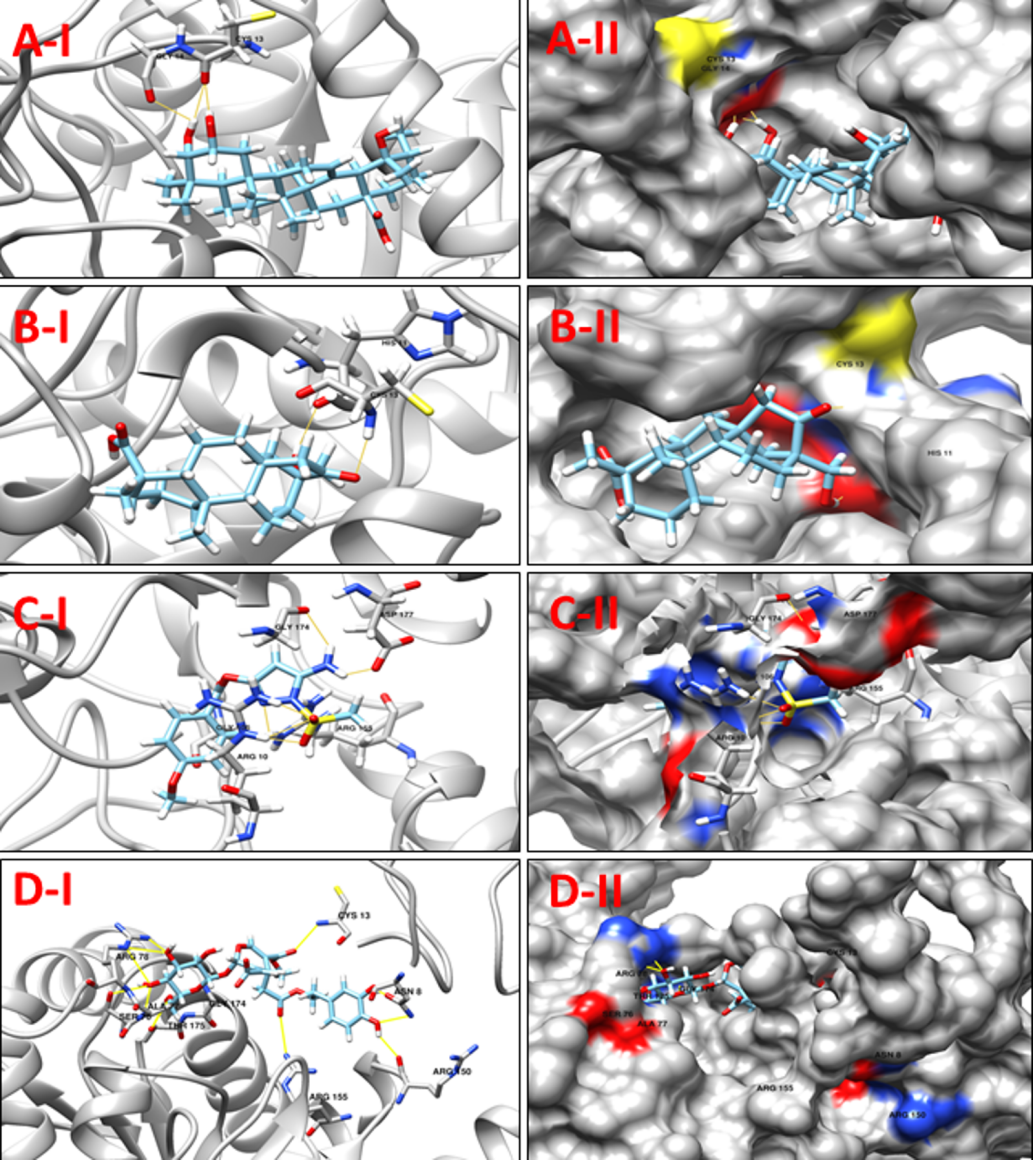
**A-1:** 3D cartoon representation of the docking analyses for the most druggable protein cavity of **NP_939345.1** (**DIP0983,** Hypothetical protein DIP0983) with Jacarandic Acid (CID 73645). **A-II:** 3D surface representation of the docking analyses for the structure of Jacarandic Acid with Hypothetical protein DIP0983. Figs **B-I, II, C-I, II** & **D-I, II** represent same information for compounds 17-hydroxyisosteviol, **ZINC00211173** and **ZINC67911471** respectively, for the same protein cavity.

**NP_939345.1** (**DIP0983,** Hypothetical protein DIP0983) is a conserved hypothetical protein. It is annotated as a possible lysine decarboxylase (LDC) in the Pfam database (PF03641) [54] due to the presence of the highly conserved PGGxGTxxE motif. Some enzymes *i*:*e* “Lonely Guy” LOG are often mis-annotated as lysine decarboxylases enzymes; it is apparently responsible for catalyzing L-lysine decarboxylation to produce the polyamine metabolite cadaverine [55]. Conversely, this annotation is not supported by any biochemical or functional data in any of the PGGxGTxxE motif containing LDC identified so far. This motif is highly conserved among a vast number of proteins with unknown function, predicted from bacterial, yeast, and plant; in *Arabidopsis thaliana*, all the genome-annotated LOG proteins are identified as LDC like proteins by protein family. Based on sequence BLAST against the PDB, LOG from *Claviceps purpurea* shares more than 30% identical residues with crystal structures of LDC-like proteins of unknown function, whose structures are already determined. Recently, lysine decarboxylase has been reported as a therapeutic target by Lohinai *et al*., 2015 for Periodontal Inflammation [56]. Here we listed 12 compounds showing good potency against our target tabulated in **Table 11**. Four of the compounds with promising docking results are shown in **Fig 12.**

**Table 11:** Compounds/Libraries name, MolDock scores and predicted hydrogen bonds for the selected molecules against NP_939345.1 (DIP0983, hypothetical protein DIP0983).

Compounds		MolDock Score	H-Bond/Residues
**Plant derived natural compounds**	Rhein	-55.7819	3/ Cys13, Leu17, Asp177
	Jacarandic Acid	-80.8294	3/ Cys13, Gly14
**Derivative of diterpenoid isosteviol**	17-hydroxyisosteviol	-95.9025	2/ His11, Cys13
	16–17 dihydroxyisosteviol	-83.7226	2/ His11, Cys13
**ZINC Compounds**	ZINC00114311	-125.423	6/ Arg10, Arg155, Gly172
	ZINC00211173	-98.6064	7/ Arg10, Gly106, Arg155, Gly174, Asp177
	ZINC01427915	-112.22	6/ Arg10, Ala77, Arg78, Gly172, Gly174
	ZINC04836994	-136.847	5/ Arg10, Gly106, Ile131, Glu132
	ZINC32004947	-146.72	5/ Arg10, Gly106, Ile131, Glu132
**ZINC NP Compounds**	ZINC67911471	-176.091	13/ Asn8, Cys13, Ser76, Ala77, Arg78 Arg150, Arg155, Gly174, Thr175
	ZINC31163223	-162.908	12/ Asn8, Arg10, His11, Arg150, Arg155, Gly172, Thr175
	ZINC04096393	-148.423	10/ Glu9, Arg10, Ala77, Arg78, Arg155, Lys156, Thr175

Among the drug-like molecule **ZINC13142972 (**1-[(2S, 3S, 4S, 5R)-3,4-dihydroxy-5-(hydroxymethyl) oxolan-2-yl]imidazo[1,2-b]pyrazole-7-carbonitrile) was predicted to show good results against two of our targets **NP_939302.1** (**glpX,** Fructose 1,6-bisphosphatase II) and **NP_939445.1** (**DIP1084,** Putative iron transport membrane protein, FecCD-family). It has been reported that at present 50% of drug molecules are either from natural source or their derivatives [57]. Interestingly, the compounds from second library of ZINC (Natural Product) showed better energy scores among all the libraries. Furthermore, from the library of natural compounds (28 molecules), Jacarandic Acid and Rhein were identified as the top ranked molecules and *in silico* analysis of the library (derivatives of diterpenoid isosteviol) suggest that compounds 16-hydroxyisosteviol, 16-hydrazonisosteviol, 17-hydroxyisosteviol, 16–17 dihydroxyisosteviol and 16-oxime, 17-hydroxyisosteviol were top ranked molecules, however, with much higher energy scores (less negative) than the top compounds from the ZINC libraries (ZINC drug-like molecules, ZINC Natural Product).

## Conclusion

We utilized a bioinformatics pipeline for determining the conserved proteome of 13 strains of *C*. *diphtheriae*, and subsequently exploit 3D structural information, resulting in a small set of prioritized putative drug/vaccine targets, of which eight proteins are pathogen-essential, non-host homologous and 15 are pathogen-essential, host-homologs. After a detailed structural comparison between host and pathogen proteins, we suggest that eight of the non -host homologs could be considered for antimicrobial chemotherapy in future studies on anti-diphtheriae drugs and vaccines. Moreover, the strategy described herein is of general nature and can also be employed to other pathogenic microorganisms.

## Supporting information

S1 Table Structural information of the Di-terpenoid Iso-steviol derivatives. (DOCX)

S2 Table Information of templates used for 8 essential non host homologous targets. (DOCX)

S3 Table Common conserved proteins with their templates. (XLS)

## Abbreviations

BLAST: Basic Local Alignment Search Tool
Cd: *Corynebacterium diphtheriae*
DNA: Deoxyribonucleic acid
LDC: Lysine decarboxylase
MVD: Molegro Virtual Docker
PDB: Protein Data Bank
RNA: Ribonucleic acid
NP: Natural Product

## References

[1] Funke G, von Graevenitz A, Clarridge JE, 3rd, Bernard KA. Clinical microbiology of coryneform bacteria. Clin Microbiol Rev. 1997;10(1):125–59. ; PubMed Central PMCID: PMCPMC172946. 8993861 10.1128/cmr.10.1.125 PMC172946

[2] Goodfellow M, Kämpfer P,. Busse HJ, Trujillo M, Suzuki KI, Ludwig W. Whitman Bergey’s manual of systematic bacteriology: Springer; 2012.

[3] Hodes HL. Diphtheria. Pediatr Clin North Am. 1979;26(2):445–59. . 379784 10.1016/s0031-3955(16)33716-6

[4] Hart PE, Lee PY, Macallan DC, Wansbrough-Jones MH. Cutaneous and pharyngeal diphtheria imported from the Indian subcontinent. Postgrad Med J. 1996;72(852):619–20. ; PubMed Central PMCID: PMCPMC2398589. 8977947 10.1136/pgmj.72.852.619 PMC2398589

[5] Wagner KS, White JM, Crowcroft NS, De Martin S, Mann G, Efstratiou A. Diphtheria in the United Kingdom, 1986–2008: the increasing role of Corynebacterium ulcerans. Epidemiol Infect. 2010;138(11):1519–30. doi: 10.1017/S0950268810001895 . 20696088 10.1017/S0950268810001895

[6] Barh D, Gupta K, Jain N, Khatri G, Leon-Sicairos N, Canizalez-Roman A, et al Conserved host-pathogen PPIs. Globally conserved inter-species bacterial PPIs based conserved host-pathogen interactome derived novel target in C. pseudotuberculosis, C. diphtheriae, M. tuberculosis, C. ulcerans, Y. pestis, and E. coli targeted by Piper betel compounds. Integr Biol (Camb). 2013;5(3):495–509. doi: 10.1039/c2ib20206a . 23288366 10.1039/c2ib20206a

[7] Perumal D, Lim CS, Sakharkar KR, Sakharkar MK. Differential genome analyses of metabolic enzymes in Pseudomonas aeruginosa for drug target identification. In Silico Biol. 2007;7(4–5):453–65. . 18391237

[8] Pizza M, Scarlato V, Masignani V, Giuliani MM, Arico B, Comanducci M, et al Identification of vaccine candidates against serogroup B meningococcus by whole-genome sequencing. Science. 2000;287(5459):1816–20. . 10710308 10.1126/science.287.5459.1816

[9] Asif SM, Asad A, Faizan A, Anjali MS, Arvind A, Neelesh K, et al Dataset of potential targets for Mycobacterium tuberculosis H37Rv through comparative genome analysis. Bioinformation. 2009;4(6):245–8. ; PubMed Central PMCID: PMCPMC2951718. 20975918 10.6026/97320630004245 PMC2951718

[10] Chong CE, Lim BS, Nathan S, Mohamed R. In silico analysis of Burkholderia pseudomallei genome sequence for potential drug targets. In Silico Biol. 2006;6(4):341–6. . 16922696

[11] Dutta A, Singh SK, Ghosh P, Mukherjee R, Mitter S, Bandyopadhyay D. In silico identification of potential therapeutic targets in the human pathogen Helicobacter pylori. In Silico Biol. 2006;6(1–2):43–7. . 16789912

[12] Sakharkar KR, Sakharkar MK, Chow VT. A novel genomics approach for the identification of drug targets in pathogens, with special reference to Pseudomonas aeruginosa. In Silico Biol. 2004;4(3):355–60. . 15724285

[13] Barh D, Kumar A. In silico identification of candidate drug and vaccine targets from various pathways in Neisseria gonorrhoeae. In Silico Biol. 2009;9(4):225–31. . 20109152

[14] Rathi B, Sarangi AN, Trivedi N. Genome subtraction for novel target definition in Salmonella typhi. Bioinformation. 2009;4(4):143–50. ; PubMed Central PMCID: PMCPMC2825597. 20198190 10.6026/97320630004143 PMC2825597

[15] Barh D, Jain N, Tiwari S, Parida BP, D'Afonseca V, Li L, et al A novel comparative genomics analysis for common drug and vaccine targets in Corynebacterium pseudotuberculosis and other CMN group of human pathogens. Chem Biol Drug Des. 2011;78(1):73–84. doi: 10.1111/j.1747-0285.2011.01118.x . 21443692 10.1111/j.1747-0285.2011.01118.x

[16] Aronov AM, Verlinde CL, Hol WG, Gelb MH. Selective tight binding inhibitors of trypanosomal glyceraldehyde-3-phosphate dehydrogenase via structure-based drug design. J Med Chem. 1998;41(24):4790–9. doi: 10.1021/jm9802620 . 9822549 10.1021/jm9802620

[17] Singh S, Malik BK, Sharma DK. Molecular modeling and docking analysis of Entamoeba histolytica glyceraldehyde-3 phosphate dehydrogenase, a potential target enzyme for anti-protozoal drug development. Chem Biol Drug Des. 2008;71(6):554–62. doi: 10.1111/j.1747-0285.2008.00666.x . 18489439 10.1111/j.1747-0285.2008.00666.x

[18] Hassan SS, Tiwari S, Guimaraes LC, Jamal SB, Folador E, Sharma NB, et al Proteome scale comparative modeling for conserved drug and vaccine targets identification in Corynebacterium pseudotuberculosis. BMC Genomics. 2014;15 Suppl 7:S3 doi: 10.1186/1471-2164-15-S7-S3 ; PubMed Central PMCID: PMCPMC4243142. 25573232 10.1186/1471-2164-15-S7-S3 PMC4243142

[19] Eswar N, Webb B, Marti-Renom MA, Madhusudhan MS, Eramian D, Shen MY, et al Comparative protein structure modeling using MODELLER. Curr Protoc Protein Sci. 2007;Chapter 2:Unit 2 9. doi: 10.1002/0471140864.ps0209s50 . 18429317 10.1002/0471140864.ps0209s50

[20] Mount DW. Using the Basic Local Alignment Search Tool (BLAST). CSH Protoc. 2007;2007:pdb top17. doi: 10.1101/pdb.top17 . 21357135 10.1101/pdb.top17

[21] Tusnady GE, Simon I. The HMMTOP transmembrane topology prediction server. Bioinformatics. 2001;17(9):849–50. . 11590105 10.1093/bioinformatics/17.9.849

[22] Laskowski RA, MacArthur MW, Moss DS and Thornton JM. PROCHECK: a program to check the stereochemical quality of protein structures. Journal of Applied Crystallography. 1993;26 Epub 291. doi: 10.1107/S0021889892009944

[23] Blom J, Albaum SP, Doppmeier D, Puhler A, Vorholter FJ, Zakrzewski M, et al EDGAR: a software framework for the comparative analysis of prokaryotic genomes. BMC Bioinformatics. 2009;10:154 doi: 10.1186/1471-2105-10-154 ; PubMed Central PMCID: PMCPMC2696450. 19457249 10.1186/1471-2105-10-154 PMC2696450

[24] Abadio AK, Kioshima ES, Teixeira MM, Martins NF, Maigret B, Felipe MS. Comparative genomics allowed the identification of drug targets against human fungal pathogens. BMC Genomics. 2011;12:75 doi: 10.1186/1471-2164-12-75 ; PubMed Central PMCID: PMCPMC3042012. 21272313 10.1186/1471-2164-12-75 PMC3042012

[25] Zhang R, Ou HY, Zhang CT. DEG: a database of essential genes. Nucleic Acids Res. 2004;32(Database issue):D271–2. doi: 10.1093/nar/gkh024 ; PubMed Central PMCID: PMCPMC308758. 14681410 10.1093/nar/gkh024 PMC308758

[26] Kanehisa M, Goto S. KEGG: kyoto encyclopedia of genes and genomes. Nucleic Acids Res. 2000;28(1):27–30. ; PubMed Central PMCID: PMCPMC102409. 10592173 10.1093/nar/28.1.27 PMC102409

[27] Magrane M, Consortium U. UniProt Knowledgebase: a hub of integrated protein data. Database (Oxford). 2011;2011:bar009 doi: 10.1093/database/bar009 ; PubMed Central PMCID: PMCPMC3070428. 21447597 10.1093/database/bar009 PMC3070428

[28] Yoon SH, Park YK, Lee S, Choi D, Oh TK, Hur CG, et al Towards pathogenomics: a web-based resource for pathogenicity islands. Nucleic Acids Res. 2007;35(Database issue):D395–400. doi: 10.1093/nar/gkl790 ; PubMed Central PMCID: PMCPMC1669727. 17090594 10.1093/nar/gkl790 PMC1669727

[29] Yu CS, Lin CJ, Hwang JK. Predicting subcellular localization of proteins for Gram-negative bacteria by support vector machines based on n-peptide compositions. Protein Sci. 2004;13(5):1402–6. doi: 10.1110/ps.03479604 ; PubMed Central PMCID: PMCPMC2286765. 15096640 10.1110/ps.03479604 PMC2286765

[30] Aguero F, Al-Lazikani B, Aslett M, Berriman M, Buckner FS, Campbell RK, et al Genomic-scale prioritization of drug targets: the TDR Targets database. Nat Rev Drug Discov. 2008;7(11):900–7. doi: 10.1038/nrd2684 ; PubMed Central PMCID: PMCPMC3184002. 18927591 10.1038/nrd2684 PMC3184002

[31] Butt AM, Nasrullah I, Tahir S, Tong Y. Comparative genomics analysis of Mycobacterium ulcerans for the identification of putative essential genes and therapeutic candidates. PLoS One. 2012;7(8):e43080 doi: 10.1371/journal.pone.0043080 ; PubMed Central PMCID: PMCPMC3418265. 22912793 10.1371/journal.pone.0043080 PMC3418265

[32] Volkamer A, Kuhn D, Rippmann F, Rarey M. DoGSiteScorer: a web server for automatic binding site prediction, analysis and druggability assessment. Bioinformatics. 2012;28(15):2074–5. doi: 10.1093/bioinformatics/bts310 . 22628523 10.1093/bioinformatics/bts310

[33] Tiwari S, da Costa MP, Almeida S, Hassan SS, Jamal SB, Oliveira A, et al C. pseudotuberculosis Phop confers virulence and may be targeted by natural compounds. Integr Biol (Camb). 2014;6(11):1088–99. doi: 10.1039/c4ib00140k . 25212181 10.1039/c4ib00140k

[34] Voigt JH, Bienfait B, Wang S, Nicklaus MC. Comparison of the NCI open database with seven large chemical structural databases. J Chem Inf Comput Sci. 2001;41(3):702–12. . 11410049 10.1021/ci000150t

[35] Wadood A, Jamal SB, Riaz M, Mir A. Computational analysis of benzofuran-2-carboxlic acids as potent Pim-1 kinase inhibitors. Pharm Biol. 2014;52(9):1170–8. doi: 10.3109/13880209.2014.880488 . 24766364 10.3109/13880209.2014.880488

[36] Thomsen R, Christensen MH. MolDock: a new technique for high-accuracy molecular docking. J Med Chem. 2006;49(11):3315–21. doi: 10.1021/jm051197e . 16722650 10.1021/jm051197e

[37] Pettersen EF, Goddard TD, Huang CC, Couch GS, Greenblatt DM, Meng EC, et al UCSF Chimera—a visualization system for exploratory research and analysis. J Comput Chem. 2004;25(13):1605–12. doi: 10.1002/jcc.20084 . 15264254 10.1002/jcc.20084

[38] Caffrey CR, Rohwer A, Oellien F, Marhofer RJ, Braschi S, Oliveira G, et al A comparative chemogenomics strategy to predict potential drug targets in the metazoan pathogen, Schistosoma mansoni. PLoS One. 2009;4(2):e4413 doi: 10.1371/journal.pone.0004413 ; PubMed Central PMCID: PMCPMC2635471. 19198654 10.1371/journal.pone.0004413 PMC2635471

[39] Crowther GJ, Shanmugam D, Carmona SJ, Doyle MA, Hertz-Fowler C, Berriman M, et al Identification of attractive drug targets in neglected-disease pathogens using an in silico approach. PLoS Negl Trop Dis. 2010;4(8):e804 doi: 10.1371/journal.pntd.0000804 ; PubMed Central PMCID: PMCPMC2927427. 20808766 10.1371/journal.pntd.0000804 PMC2927427

[40] Shanmugham B, Pan A. Identification and characterization of potential therapeutic candidates in emerging human pathogen Mycobacterium abscessus: a novel hierarchical in silico approach. PLoS One. 2013;8(3):e59126 doi: 10.1371/journal.pone.0059126 ; PubMed Central PMCID: PMCPMC3602546. 23527108 10.1371/journal.pone.0059126 PMC3602546

[41] Folador EL, de Carvalho PV, Silva WM, Ferreira RS, Silva A, Gromiha M, et al In silico identification of essential proteins in Corynebacterium pseudotuberculosis based on protein-protein interaction networks. BMC Syst Biol. 2016;10(1):103 doi: 10.1186/s12918-016-0346-4 ; PubMed Central PMCID: PMCPMC5097352. 27814699 10.1186/s12918-016-0346-4 PMC5097352

[42] Wadood A, Riaz M, Jamal SB, Shah M. Interactions of ketoamide inhibitors on HCV NS3/4A protease target: molecular docking studies. Mol Biol Rep. 2014;41(1):337–45. doi: 10.1007/s11033-013-2867-x . 24234753 10.1007/s11033-013-2867-x

[43] Horecker BL, Melloni E, Pontremoli S. Fructose 1,6-bisphosphatase: properties of the neutral enzyme and its modification by proteolytic enzymes. Adv Enzymol Relat Areas Mol Biol. 1975;42:193–226. . 236638 10.1002/9780470122877.ch4

[44] Wright SW, Carlo AA, Carty MD, Danley DE, Hageman DL, Karam GA, et al Anilinoquinazoline inhibitors of fructose 1,6-bisphosphatase bind at a novel allosteric site: synthesis, in vitro characterization, and X-ray crystallography. J Med Chem. 2002;45(18):3865–77. . 12190310 10.1021/jm010496a

[45] Sassetti CM, Rubin EJ. Genetic requirements for mycobacterial survival during infection. Proc Natl Acad Sci U S A. 2003;100(22):12989–94. doi: 10.1073/pnas.2134250100 ; PubMed Central PMCID: PMCPMC240732. 14569030 10.1073/pnas.2134250100 PMC240732

[46] Gopal B, Haire LF, Cox RA, Jo Colston M, Major S, Brannigan JA, et al The crystal structure of NusB from Mycobacterium tuberculosis. Nat Struct Biol. 2000;7(6):475–8. doi: 10.1038/75876 . 10881194 10.1038/75876

[47] Brogan AP, Verghese J, Widger WR, Kohn H. Bismuth-dithiol inhibition of the Escherichia coli rho transcription termination factor. J Inorg Biochem. 2005;99(3):841–51. doi: 10.1016/j.jinorgbio.2004.12.019 . 15708806 10.1016/j.jinorgbio.2004.12.019

[48] Yates JL, Arfsten AE, Nomura M. In vitro expression of Escherichia coli ribosomal protein genes: autogenous inhibition of translation. Proc Natl Acad Sci U S A. 1980;77(4):1837–41. ; PubMed Central PMCID: PMCPMC348603. 6445562 10.1073/pnas.77.4.1837 PMC348603

[49] Davies C, Ramakrishnan V, White SW. Structural evidence for specific S8-RNA and S8-protein interactions within the 30S ribosomal subunit: ribosomal protein S8 from Bacillus stearothermophilus at 1.9 A resolution. Structure. 1996;4(9):1093–104. . 8805594 10.1016/s0969-2126(96)00115-3

[50] Berkovitch F, Nicolet Y, Wan JT, Jarrett JT, Drennan CL. Crystal structure of biotin synthase, an S-adenosylmethionine-dependent radical enzyme. Science. 2004;303(5654):76–9. doi: 10.1126/science.1088493 ; PubMed Central PMCID: PMCPMC1456065. 14704425 10.1126/science.1088493 PMC1456065

[51] Javid-Majd F, Yang D, Ioerger TR, Sacchettini JC. The 1.25 A resolution structure of phosphoribosyl-ATP pyrophosphohydrolase from Mycobacterium tuberculosis. Acta Crystallogr D Biol Crystallogr. 2008;64(Pt 6):627–35. doi: 10.1107/S0907444908007105 ; PubMed Central PMCID: PMCPMC2631106. 18560150 10.1107/S0907444908007105 PMC2631106

[52] Gutmann S, Haebel PW, Metzinger L, Sutter M, Felden B, Ban N. Crystal structure of the transfer-RNA domain of transfer-messenger RNA in complex with SmpB. Nature. 2003;424(6949):699–703. doi: 10.1038/nature01831 . 12904796 10.1038/nature01831

[53] Finn RD, Bateman A, Clements J, Coggill P, Eberhardt RY, Eddy SR, et al Pfam: the protein families database. Nucleic Acids Res. 2014;42(Database issue):D222–30. doi: 10.1093/nar/gkt1223 ; PubMed Central PMCID: PMCPMC3965110. 24288371 10.1093/nar/gkt1223 PMC3965110

[54] Bateman A, Birney E, Cerruti L, Durbin R, Etwiller L, Eddy SR, et al The Pfam protein families database. Nucleic Acids Res. 2002;30(1):276–80. ; PubMed Central PMCID: PMCPMC99071. 11752314 10.1093/nar/30.1.276 PMC99071

[55] Dzurova L, Forneris F, Savino S, Galuszka P, Vrabka J, Frebort I. The three-dimensional structure of "Lonely Guy" from Claviceps purpurea provides insights into the phosphoribohydrolase function of Rossmann fold-containing lysine decarboxylase-like proteins. Proteins. 2015;83(8):1539–46. doi: 10.1002/prot.24835 . 26010010 10.1002/prot.24835

[56] Lohinai Z, Keremi B, Szoko E, Tabi T, Szabo C, Tulassay Z, et al Biofilm lysine Decarboxylase, a New Therapeutic Target for Periodontal Inflammation. J Periodontol. 2015:1–15. doi: 10.1902/jop.2015.140490 . 26110450 10.1902/jop.2015.140490

[57] Veeresham C. Natural products derived from plants as a source of drugs. J Adv Pharm Technol Res. 2012;3(4):200–1. doi: 10.4103/2231-4040.104709 ; PubMed Central PMCID: PMCPMC3560124. 23378939 10.4103/2231-4040.104709 PMC3560124

