# Supplementary material for: An integrative in-silico approach for therapeutic target identification in the human pathogen Corynebacterium diphtheriae
Source: PLoS One. 2017 Oct 19;12(10):e0186401. doi: 10.1371/journal.pone.0186401 (PMC5648181; doi:10.1371/journal.pone.0186401)
Supplement: S1 Table — (DOCX) [file pone.0186401.s001.docx]

|  | Structure | code |  |
| --- | --- | --- | --- |
| 1 |  | Iso  M.wt=318g | Reported |
| 2 |  | HIS/IOL  M.wt =320 g | Reported |
| 3 |  | IO  Mwt=333 g | Reported |
| 4 |  | IL  Mwt=334 g | Reported |
| 5 |  | ID  Mwt =498 g  Showed very good results in a cancer line cells. | Reported |
| 6 |  | IB  Mwt = 466 g | New |
| 7 |  | OLB  Mwt = 468 g | New |
| 8 |  | IOB  Mwt = 481 g | New |
| 9 |  | ES/ST  Mwt=318 g | Reported |
| 10 |  | EB  Mwt = 466 g | New |
| 11 |  | EP  Mwt = 334 g | Reported |
| 12 |  | IBE  Mwt = 408 g | New |
| 13 |  | HBE  Mwt = 410 g | New |
| 14 |  | OBE  Mwt = 423 g | New |
| 15 |  | IH  Mwt = 351 g | Reported |
| 16 |  | ID4  Mwt = 332 g | Reported |
| 17 |  | ID3  Mwt = 372 g | New |
| 18 |  | ID2  Mwt=453 g | New |
| 19 |  | ID7  Mwt = 514 | New |
| 20 |  | ID8  Mwt = 469 g | New |
| 21 |  | ID9  Mwt = 348 g | New |
| 22 |  | HO  Mwt= 334 g | Reported |
| 23 |  | IH2  Mwt= 336 g | Reported |
| 24 |  | HIO  Mwt =349 g | Reported |
| 25 |  |  |  |
| 26 |  |  |  |
| 27 |  | IDB4 |  |
| 28 |  | IDB3 |  |
