# Supplementary material for: An integrative in-silico approach for therapeutic target identification in the human pathogen Corynebacterium diphtheriae
Source: PLoS One. 2017 Oct 19;12(10):e0186401. doi: 10.1371/journal.pone.0186401 (PMC5648181; doi:10.1371/journal.pone.0186401)
Supplement: S2 Table — (DOCX) [file pone.0186401.s002.docx]

| **Target** | **Template** | **Chain** | **Identity** | **e-value** | **Coverage** |
| --- | --- | --- | --- | --- | --- |
| NP_939692.1, **nusB (**Transcription antitermination protein NusB**)** | 1EYV | B | 41% | 7e-29 | 78% |
| NP_939612.1, **hisE (**Phosphoribosyl-ATP pyrophosphatase**)** | 1Y6X | A | 60% | 2e-33 | 100% |
| NP_939445.1, **DIP1084 (**Putative iron transport membrane protein, FecCD-family**)** | 2NQ2 | B | 37% | 5e-52 | 98% |
| NP_939345.1, **DIP0983 (**Hypothetical protein DIP0983**)** | 1WEK | F | 52% | 4e-56 | 78% |
| NP_939302.1, **glpX (**Fructose 1,6-bisphosphatase II**)** | 1NI9 | A | 45% | 1e-90 | 92% |
| NP_939123.1, **smpB (**SsrA-binding protein**)** | 1P6V | A | 49% | 2e-43 | 86% |
| NP_938900.1, **rpsH (**30S ribosomal protein S8**)** | 1SEI | B | 59% | 2e-49 | 98% |
| NP_938502.1, **bioB (**Biotin synthase**)** | 1R30 | B | 37% | 8e-60 | 84% |
